# Resonant model—A new paradigm for modeling an action potential of biological cells

**DOI:** 10.1371/journal.pone.0216999

**Published:** 2019-05-22

**Authors:** Sucheta Sehgal, Nitish D. Patel, Avinash Malik, Partha S. Roop, Mark L. Trew

**Affiliations:** 1 Department of Electrical and Computer Engineering, The University of Auckland, Auckland 1010, New Zealand; 2 Auckland Bioengineering Institute, The University of Auckland, Auckland 1010, New Zealand; University of California Riverside, UNITED STATES

## Abstract

Organ level simulation of bioelectric behavior in the body benefits from flexible and efficient models of cellular membrane potential. These computational organ and cell models can be used to study the impact of pharmaceutical drugs, test hypotheses, assess risk and for closed-loop validation of medical devices. To move closer to the real-time requirements of this modeling a new flexible Fourier based general membrane potential model, called as a Resonant model, is developed that is computationally inexpensive. The new model accurately reproduces non-linear potential morphologies for a variety of cell types. Specifically, the method is used to model human and rabbit sinoatrial node, human ventricular myocyte and squid giant axon electrophysiology. The Resonant models are validated with experimental data and with other published models. Dynamic changes in biological conditions are modeled with changing model coefficients and this approach enables ionic channel alterations to be captured. The Resonant model is used to simulate entrainment between competing sinoatrial node cells. These models can be easily implemented in low-cost digital hardware and an alternative, resource-efficient implementations of sine and cosine functions are presented and it is shown that a Fourier term is produced with two additions and a binary shift.

## Introduction

Computer models of electrical function in excitable cells can be used to conduct pharmaceutical drug testing, assess the risk of adverse health outcomes, plan treatments and do basic science investigations [1]. The goal is to parameterize models such that organ-level patient-specific behaviors can be studied [2]. However, an emerging application is also toward functional and formal validation of medical devices [3].

At the core of organ models are cellular membrane models describing the electrophysiology of constituent excitable cells. Many of these cell models are traced to the pioneering work of Hodgkin and Huxley [4] that quantified ion currents and the action potential of nerve axons. Subsequently, many detailed electrophysiology models [2, 5, 6], reduced electrophysiology models [2], generic models [7, 8] and phenomenological models [7] have been developed. Such models are useful for testing and generating hypotheses that are otherwise difficult to address experimentally, and make computer modeling an indispensable part of biological systems research [1].

The detailed electrophysiology models may include 30-100 variables and tens to hundreds of coupled non-linear differential equations [5, 9]. The equations include computationally expensive functions such as exponents, logarithms, and exponentiation to non-integer powers. In recent years, there has been growing and relatively economic access to high-performance computing resources, enabling simulations with more biophysical detail and higher throughput. However, in spite of these resources, it remains intractable to solve, for example, 1 second of cardiac organ activity in near real-time. Therefore, alternate approaches are essential if models are to be useful for functional and formal validation of medical devices.

There are a number of investigations that have developed simplified models to reproduce action potentials from different classes of excitable biological cells [7, 8, 10–12]. However, not all these approaches are best suited to real-time execution or formal analysis. To address this, we have developed a general resonant model (RM) framework for modeling action potentials. The key features of the RM are that it is: (1) particularly amenable to parallel execution in computer hardware; (2) suitable for large-scale spatial simulations and formal analysis; and (3) the RM can be easily adapted to suit any observed behavior by modifying the underline behavioral equations. However, these features are only useful if the RM can be parameterized to capture varied electrophysiology and dynamic behavior. Here we show the RM reproducing action potential (AP) morphology of sinoatrial (SA) node cells and perturbations in the signals due to alterations in ion channel behavior. The RM framework is also shown to function effectively for modeling APs in nerve axons and cardiac myocytes.

## Methods

**Fig 1:**
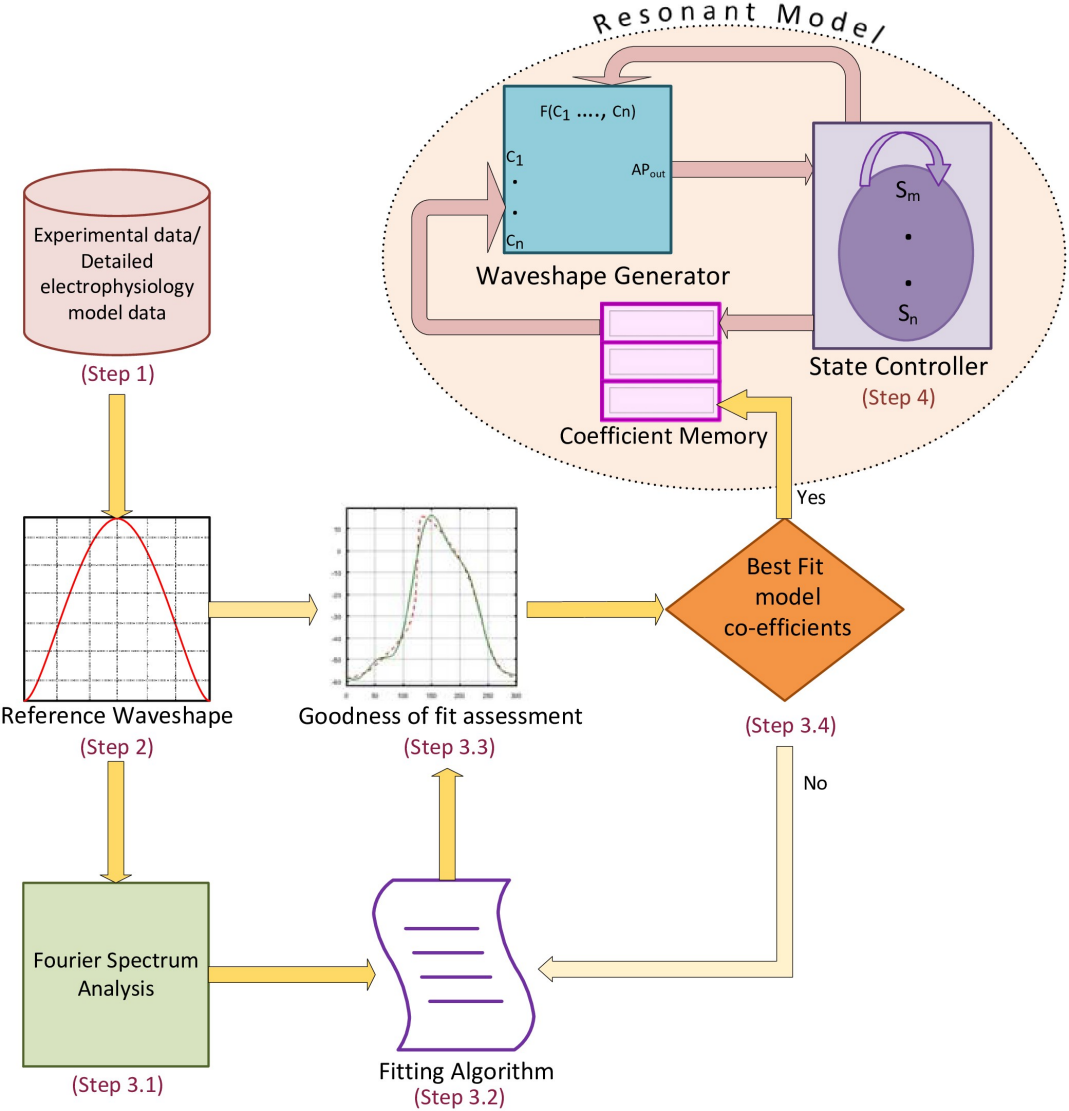
The modeling workflow. Schematic illustration of the reproduction of a biological cell action potential by the Resonant Model. The model coefficients are determined with the fitting algorithm that provides the best match for the reference action potential waveshape with a minimum least square error. The state controller is designed to respond to the changes in the normal operating conditions of a cell.

The methodology of developing the resonant model (RM) (sketched in Fig 1) is described in the following steps:

Step 1 Collect electrophysiological time domain data-sets.
Step 2 Scan the data-sets to identify several contiguous action potentials that constitute a reference set. Extract this set for subsequent use.
Step 3 Use an iterative Levenberg-Marquardt algorithm to obtain best fit model coefficients for waveshape generator.
  3.1 Generate initial Resonant model (Fourier) coefficients
  3.2 Apply fitting algorithm
  3.3 Make goodness of fit assessment and the selection of number of harmonics of the finite Fourier series
  3.4 Repeat 2, if required
Step 4 Design a state controller for changing the model parameters with the change in the operating conditions.

### Reference action potential waveshapes

As shown in Fig 1, the first step in the development of the RM is the choice of detailed electrophysiological models of different cell types as references for the determination of RM coefficients. Cardiac cellular literature details many bio-physical models with varying complexity. Although multiple models may target a specific cell type, each of their action potentials differs and pose additional challenges in selecting one over the other [13–15]. For example, the different mathematical models of human ventricular cells that have been published over the past two decades, [12, 16–20], tend to show different action potential characteristics. Therefore, the choice of the reference electrophysiology model was based on the consistency of the experimental data (cell type and species) underlying the parameterization of the cell model and on the inclusion of various ionic currents in the model. In addition, the selected models should result in simulations that exhibit stability in period and amplitude over several cycles. Based on this analysis the reference model chosen for the human ventricular cell is [20], human sinoatrial node (SAN) cell is [21], and for rabbit SAN cell is [22]. This is the second step of the modeling workflow in Fig 1.

### Fourier series approach

**Fig 2:**
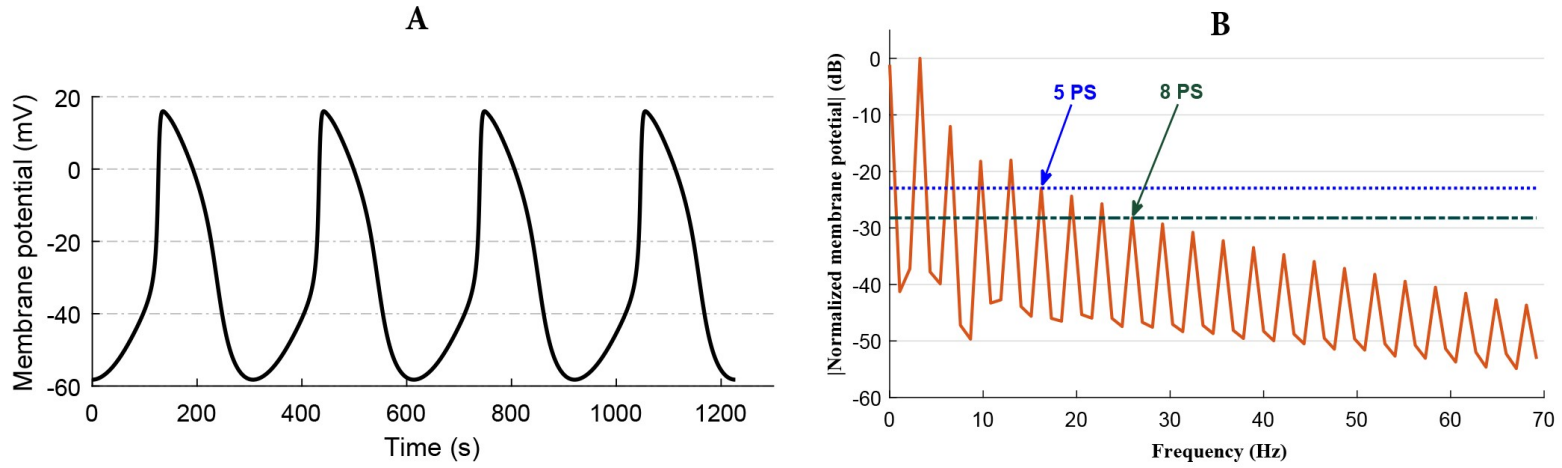
Rabbit sinoatrial node action potential waveshape and it’s frequency spectrum. A: Kurata et al.’s model-generated behavior of spontaneous APs [22]. B: Single sided amplitude spectrum of the AP waveshape generated from Kurata et al.’s model [22] output. 5 PS represents the width of the frequency spectrum covered by the Fourier series in the fifth partial sum, and 8 PS represents the width of the frequency spectrum covered by the Fourier series model in the eighth partial sum.

There exist many mathematical techniques which make it possible to define an arbitrary function of time as an infinite series. For periodic and quasi-periodic phenomena as demonstrated in the cardiac conduction and other biological systems, the Fourier series is particularly suited [23]. Therefore, the Fourier series has been used in our attempt to reproduce and quantify the electrical behavior of biological cells through a simplified and uniform mathematical model structure. The electrical pulse output *V*(*t*) of a biological cell as a result of movement of ions across the cell membrane satisfies Dirichlet’s conditions [23] and can be expressed with the trigonometric series in Eq (1).

$$\begin{array}{c} \text{V}\left(t\right)=a_{0}+\sum_{i=1}^{\infty }\left(a_{i}\;\text{cos}\frac{2\pi i}{T}t+b_{i}\;\text{sin}\frac{2\pi i}{T}t\right) \end{array}$$ (1)

where, *T* is the length of the interval (period) over which *V*(*t*) is considered; *a*_0_, *a*_*i*_, and *b*_*i*_ are Fourier coefficients; *a*_0_ represents the mean value of the function. The fundamental angular frequency is $\omega =\frac{2\pi }{T}$. As observed in the frequency spectrum of the rabbit SAN AP (Fig 2B), the magnitude of the coefficients of the higher harmonics decreases with frequency so that a given function can be closely approximated by only the first few terms of the series. The Fourier series representation of an action potential of a cell can be written as a finite number of terms as in Eq (2). As per the sampling theorem for a periodic function, 2*n* + 1 beats were taken of the reference AP waveshape to obtain a Fourier series of the desired degree of accuracy.

$$\begin{array}{c} \text{V}\left(t\right)=a_{0}+\sum_{i=1}^{n}\left(a_{i}\;\text{cos}\left(i\omega t\right)+b_{i}\;\text{sin}(i\omega t)\right) \end{array}$$ (2)

where, *a*_0_, *a*_*i*_ and *b*_*i*_ are fourier co-efficients. By combining sine and cosine terms using well-known trigonometric identities Eq (2) can be rewritten as Eq (3):

$$\begin{array}{c} \text{V}\left(t\right)=a_{0}+\sum_{i=1}^{n}c_{i}\;\text{cos}\left(i\omega t-\phi_{i}\right) \end{array}$$ (3)

where, $c_{i}=\sqrt{a_{i}^{2}+b_{i}^{2}}$, and $\phi_{i}=\tan^{-1}(\frac{b_{i}}{a_{i}})$.

These Fourier coefficients, *a*_0_, *c*_*i*_, *ϕ*_*i*_, will change with the waveshape and hence would constitute the parameters of a cell model. For a model that uses these coefficients to be useful, the coefficients must be dynamic. Modifying Eqs (3) to (4) achieves our modelling objective. Here, *γ* represents a variation in the operating condition of a cell, such as current blockage level, and influences the determination of the model parameters.

$$\begin{array}{c} \text{V}\left(t,\gamma \right)=a_{0}\left(\gamma \right)+\sum_{i=1}^{n}c_{i}\left(\gamma \right)\;\text{cos}\left(i\omega t-\phi_{i}\left(\gamma \right)\right) \end{array}$$ (4)

#### Frequency spectrum analysis and fitting algorithm

The spectrum analysis of the biological cell’s AP waveshape usually results in several tens of harmonics. Our proposed implementation would benefit from fewer harmonics, and hence it is imperative to reduce the number of harmonics significantly. The single-sided normalized amplitude spectrum of rabbit SAN AP (Fig 2A) generated from Kurata et al. model [22] is presented in Fig 2B. Of the 21 harmonics shown in the figure, only the first few harmonics are large enough to contribute significantly towards the AP waveshape. As shown in the figure, the fifth harmonic is the highest harmonic with an amplitude less than the 10% of the fundamental. The eighth harmonic is the highest harmonic with an amplitude less than 5% of the maximum amplitude. However, reconstituting the waveshape using a reduced number of harmonics introduces inaccuracies in the form of ripples and deviation from the reference waveshape.

To reduce these inaccuracies, we calculate the Fourier coefficients using an iteratively reweighted least squares algorithm. To overcome the confined abilities of simple fitting algorithms [24], and to increase the probability of finding the set of the FS coefficients within the global minimum region of the root mean square error, the values of the FS coefficients were used as starting points in the fitting algorithm have been calculated from the frequency spectrum of the biological cell AP waveshape (Step 3.1 in Fig 1). We use a robust Levenberg-Marquardt fitting algorithm [25] with three weight functions: bisquare weights (*w*_*B*_), huber weights (*w*_*H*_), and logistic weights (*w*_*L*_) as described in Eqs (5), (6) and (7), respectively (Step 3.2 in Fig 1).

$$\begin{array}{c} w_{B}=\{\begin{array}{ccc} {\left(1-{\left(\frac{u}{d}\right)}^{2}\right)}^{2} & \text{if} & |u|\leq d\\ 0 & \text{if} & |u|>d \end{array} \end{array}$$ (5)

$$\begin{array}{c} w_{H}=\{\begin{array}{ccc} 1 & \text{if} & |u|<d\\ \frac{d}{\left|u\right|} & \text{if} & |u|\geq d \end{array} \end{array}$$ (6)

$$\begin{array}{c} w_{L}=\{\frac{\text{tanh}(\frac{u}{d})}{\frac{u}{d}} \end{array}$$ (7)

Here, *d* is the tuning constant and it’s default value for *w*_*B*_ is 4.685, for *w*_*H*_ is 1.345, and for *w*_*L*_ is 1.205; *u* defines the adjusted residuals and is evaluated as $\frac{\text{residuals}}{\sqrt{(1-\text{diag}\left(\hat{H}\right)}}$; $\hat{H}$ is the Hessian matrix and is evaluated as *J*(*J*^*T*^ *J*)^−1^*J*^*T*^; *J* is the Jacobian matrix.

Different values of the tuning constant alter the shape of the weighting function and this is a significant contributor to the performance of an estimator [26]. The choice of the tuning constant was based on the associated minimum root mean square error. The final selection of the coefficients will be based on the metrics of the root mean square error and the adjusted R-squared value (Step 3.3, 3.4 in Fig 1). We have selected the number of harmonics of finite FS, to represent an AP waveshape, based on the adjusted R-squared value. The minimum number of harmonics that capture more than 99% of the reference AP variability (adjusted R-squared value higher than 0.99) are included in the FS. The best-fit model coefficients of the finite FS are stored in the coefficient memory of the RM cell.

### A Resonant model design compatible with hardware

**Fig 3:**
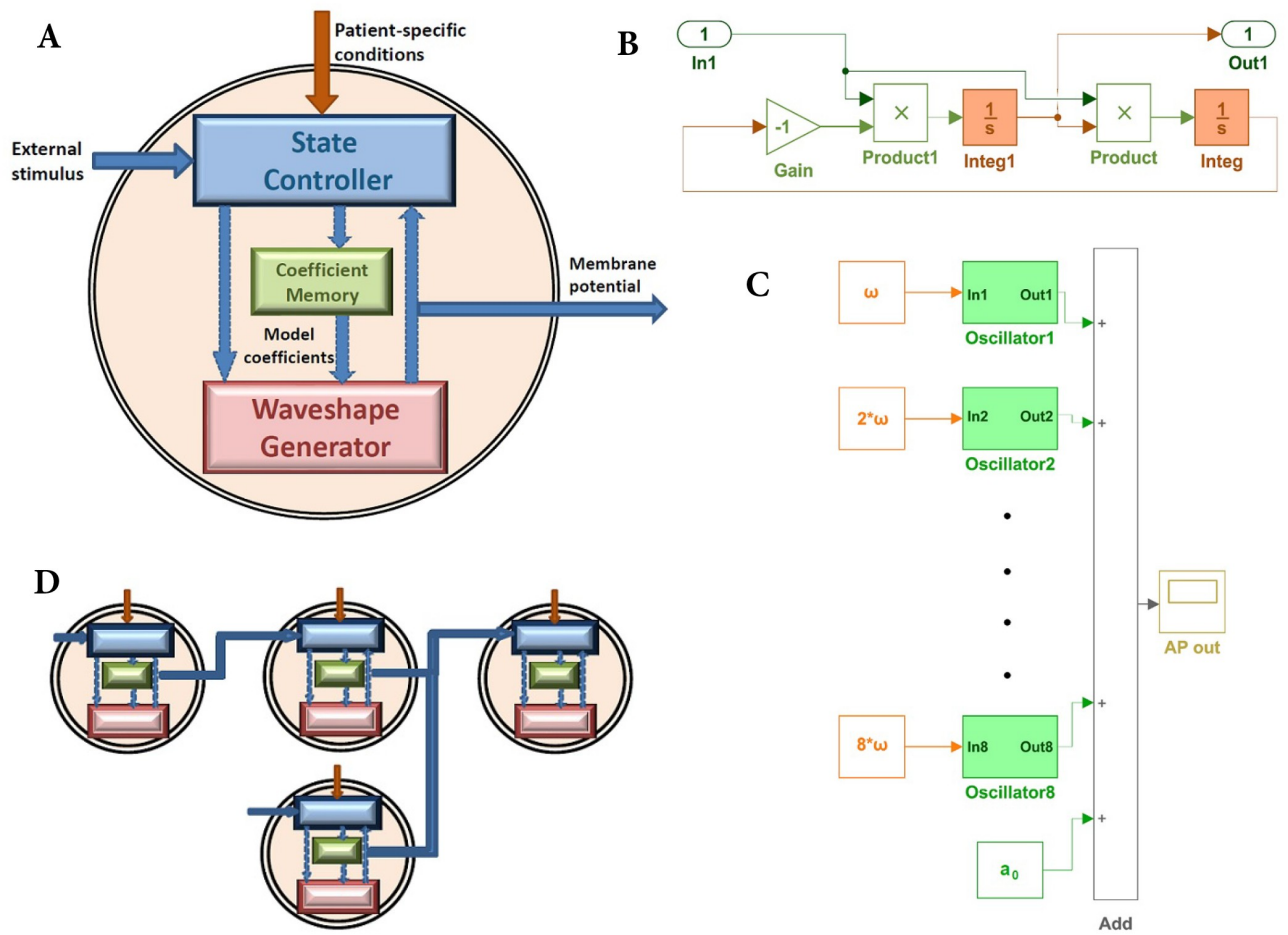
Resonant model of a biological cell. A: Schematic representation of a resonant model. B: An oscillator implemented with two integrators in series generating sine term and cosine term of the same frequency simultaneously. C: The waveshape generator implemented in SIMULINK with eight Fourier terms. D: Pictorial representation of the compositional property (series and parallel connections) of the RM cells.

The set of Fourier coefficients together with sinusoidal generators is a perpetual oscillator. Fig 3A shows the diagram of a RM of a biological cell. The waveshape generator (set of sinusoidal generators), state controller and coefficient memory constitute a RM. The waveshape generator reproduces the action potential waveshape of a biological cell. Since sine and cosine terms are computationally expensive, an alternative implementation would be valuable. We hypothesize that a sinusoidal generator constructed using two integrators in series in a closed loop offers significant implementation advantages. Fig 3B shows a sinusoidal generator which is encapsulated as a single entity and called an oscillator. The frequency of this oscillator is modified by applying a suitable input at In1.

Although each oscillator is capable of producing both sine and cosine waveshapes simultaneously, a resource-saving alternative can be constructed. Here, each of the integrators can be suitably initialized such that the output generates *c*_*i*_ cos(*iωt* − *ϕ*_*i*_). There are as many oscillators as there are terms in the FS (Eq (4)). All these oscillators together with a constant term form a waveshape generator. The structure of the waveshape generator corresponding to eight Fourier terms built in SIMULINK is shown in Fig 3C. The model consisted of eight oscillators and a constant. The initial values for all the integrators are stored in the cell memory. The waveshape generator is capable of oscillating and producing AP waveshape without an input which is the characteristic of autorhythmic cells in the cardiac conduction system. A state controller is designed to incorporate various effects and properties exhibited by the biological cells. The parameters of the state controller are defined by the type of the biological cell (autorhythmic cell, cardiomyocyte, or neuron). These parameters determine the response to an external stimulus to a cell which can be from neighboring cells or the impact of drugs, and so forth. The state controller is discussed in further detail in the following sections. RM cells can be connected together to form a single or multidimensional network of cells as shown in Fig 3D. The net ionic flow from the neighboring cells is translated into an effective potential using a coupling coefficient.

### Mutual entrainment

**Fig 4:**
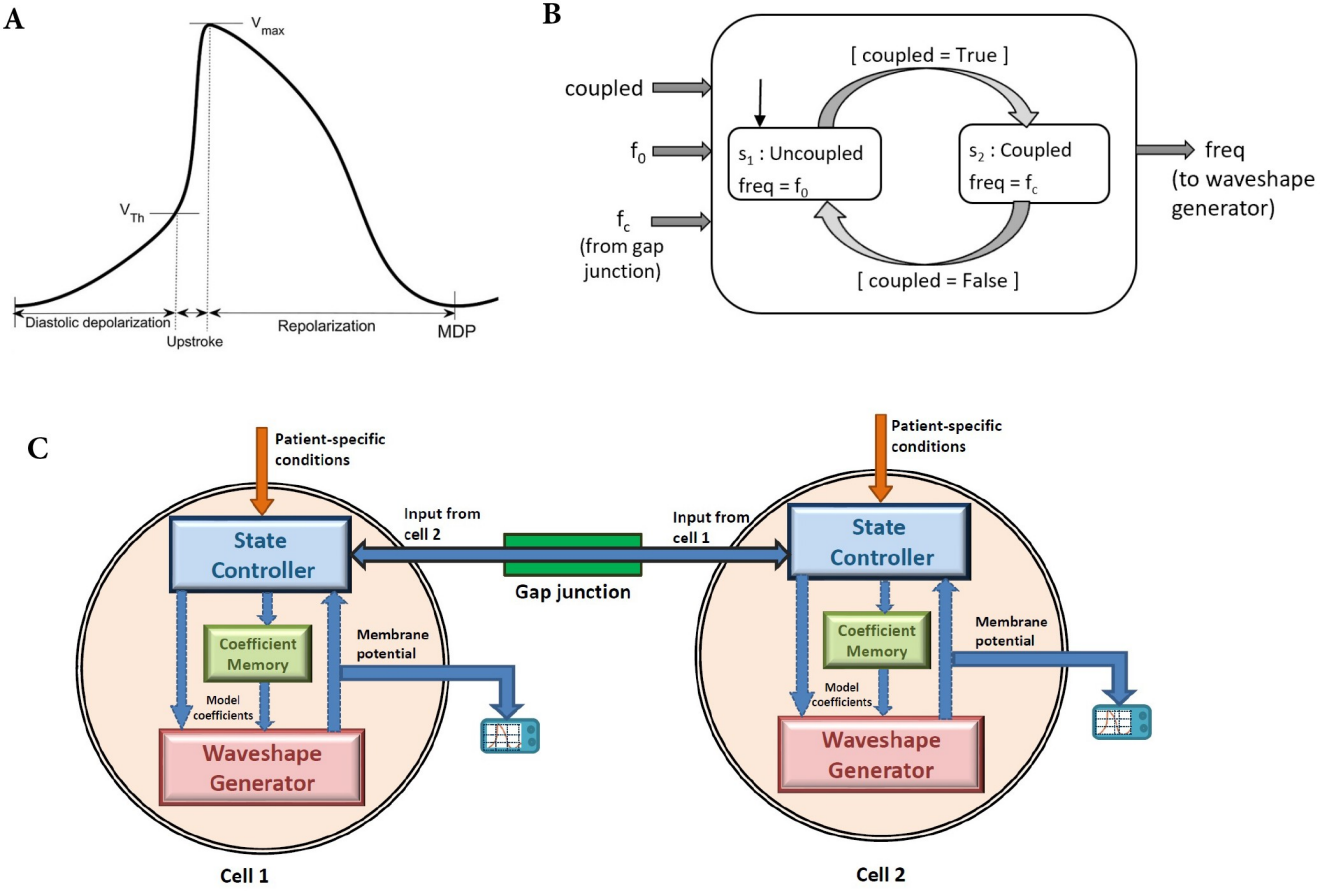
Coupling between two Resonant model sinoatrial node cells. A: Parameters of the action potential of sinoatrial node cell. MDP is a maximal diastolic potential, V_max_ is the maximum potential, and V_Th_ is the threshold potential. B: State controller of the Resonant model of a sinoatrial node cell. f_0_ is the intrinsic beating frequency of a cell and f_c_ is the entrained frequency C: Diagram of the two Resonant model cells (Cell 1 and Cell 2) coupled with a gap junction.

Experimentally obtained AP waveshapes from isolated SAN cells exhibit significant variations in AP characteristics, including intrinsic beating frequency (f_0_) [27], [28], [29]. Not withstanding this, the myocardium still undergoes robust synchronous rhythmic contractions. These contractions occur as a consequence of mutual entrainment of SAN cells [30], [31]. Fig 4A shows a typical SAN AP. MDP is a maximal diastolic potential, V_max_ is the maximum potential attained during depolarization. V_Th_ is the nominal threshold potential at which depolarization accelerates (upstroke). These three salient membrane potentials constitute the boundaries of three phases in the SAN AP waveshape. The three phases are labeled as diastolic depolarization, upstroke and, repolarization. Verheijck et al. [32] have reported that the modulation of the cycle length is largely dependent on the modulation of the diastolic depolarization phase, especially diastolic depolarization time (DDT). We introduce this capability to our model by adapting the beating frequency via the state controller (Fig 4B) and the gap junction. When the two RM cells are coupled (with coupling strength in excess of critical conductance), the state controllers of both cells make a transition to the coupled state. in which the cells beat in accordance with the equation Eq (8). In this state, the synchronized beating frequency (f_c_) is passed to the waveshape generator. The modulation in the beating frequency is calculated using Eq (8) [32]. This equation models the coupling between two cells indicated by the gap junction in Fig 4C.

$$\begin{array}{c} {\text{DDT}}_{\text{c}}=\frac{2*({\text{DDT}}_{\text{f}}*{\text{DDT}}_{\text{s}})}{{\text{DDT}}_{\text{f}}+{\text{DDT}}_{\text{s}}} \end{array}$$ (8)

DDT is defined as the time required for the potential to rise from the maximum diastolic potential to a value 20 mV positive to this potential. DDT_s_ and DDT_f_ are the DDT for the intrinsic uncoupled beating of the slower and the faster cell, respectively. DDT_c_ is the DDT for the coupled synchronized beating of the cell pair.

To model the entrainment in a network of cells, the Eq (8) is extended to Eq (9). The number of cells present in a network is represented by *n*.

$$\begin{array}{c} \frac{1}{{\text{DDT}}_{\text{c}}}=\frac{1}{\text{n}}\sum_{\text{i}=1}^{\text{n}}\frac{1}{{\text{DDT}}_{\text{i}}} \end{array}$$ (9)

### Parametrization of the Resonant model

**Fig 5:**
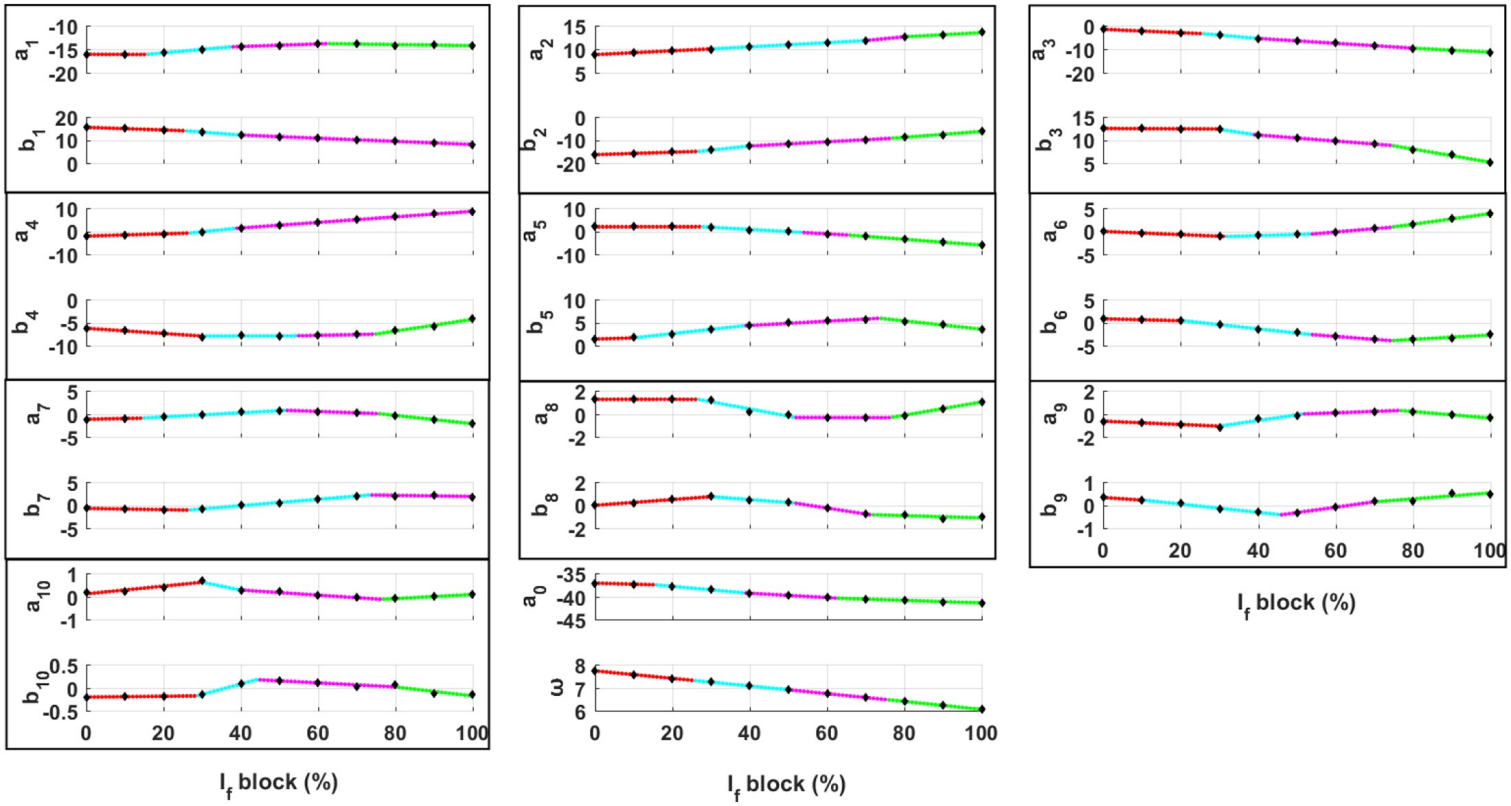
Minimum root mean squared error piecewise linear fits to the coefficients of the Resonant model (RM) of a human sinoatrial node cell at different levels of *I*_*f*_ blockage. The black diamonds represent the RM coefficients obtained after fitting to AP waveshapes of Fabbri et al. model for specific percentage values of *I*_*f*_ blockage. Dotted lines represent the piecewise linear fits to the coefficients. Different colors represent different linear equation fits. Coefficients of every oscillator in the RM are placed together in a black rectangular box. *ω* represents the fundamental frequency.

In this section, we explain the methodology that quantitatively incorporates the electrophysiology of ionic current perturbations. The Fabbri et al. model [21] of human SAN was simulated by varying the maximum funny current (*I*_*f*_) conductance *g*_*f*_ with modulating parameter *γ* lying between 0 and 1 such that $g_{f}^{\prime }=\gamma *g_{f}$. Eleven different AP waveshapes were obtained corresponding to eleven equally spaced values of *γ*. The coefficients of RM (with ten oscillators) for each of the eleven APs were obtained using the methodology shown in Fig 1. Black diamonds in Fig 5 show the variations in coefficients as a function of *γ*. It can be clearly seen that the coefficients do not exhibit a monotonic change. Therefore, we attempted to obtain closed-form equations that describe the change in coefficients as a function of *γ*: *a*_0_(*γ*), *b*_1_(*γ*) and so on. With these equations, we can construct an AP waveshape at an arbitrary *γ* between 0 and 1.

Six different levels of complexity were evaluated, and their expressions are defined in Table 1. PL2, PL3, and PL4 use two, three, and four linear equations, respectively. PC2 is a fit with two cubic equations. PO7 and PO8 are the polynomial fits of order seven and eight, respectively. The optimal locations of breakpoints (*r*_1_, *r*_2_, and *r*_3_) were determined using least squares.

**Table 1:** General expression of various fitting functions.

Fit-type	Equation
PL2	$K\left(\gamma \right)=\{\begin{array}{ccc} p_{10}+p_{11}*\gamma & \text{if} & 0<\gamma \leq r_{1}\\ p_{20}+p_{21}*\gamma & \text{if} & r_{1}<\gamma \leq 1 \end{array}$
PL3	$K\left(\gamma \right)=\{\begin{array}{ccc} p_{10}+p_{11}*\gamma & \text{if} & 0<\gamma \leq r_{1}\\ p_{20}+p_{21}*\gamma & \text{if} & r_{1}<\gamma \leq r_{2}\\ p_{30}+p_{31}*\gamma & \text{if} & r_{2}<\gamma \leq 1 \end{array}$
PL4	$K\left(\gamma \right)=\{\begin{array}{ccc} p_{10}+p_{11}*\gamma & \text{if} & 0<\gamma \leq r_{1}\\ p_{20}+p_{21}*\gamma & \text{if} & r_{1}<\gamma \leq r_{2}\\ p_{30}+p_{31}*\gamma & \text{if} & r_{2}<\gamma \leq r_{3}\\ p_{40}+p_{41}*\gamma & \text{if} & r_{3}<\gamma \leq 1 \end{array}$
PC2	$K\left(\gamma \right)=\{\begin{array}{ccc} p_{10}+\sum_{d=1}^{3}\left(p_{1d}*\gamma^{d}\right) & \text{if} & 0<\gamma \leq r_{1}\\ p_{20}+\sum_{d=1}^{3}\left(p_{2d}*\gamma^{d}\right) & \text{if} & r_{1}<\gamma \leq 1 \end{array}$
PO7	$K\left(\gamma \right)=p_{0}+\sum_{d=1}^{7}\left(p_{d}*\gamma^{d}\right)$
PO8	$K\left(\gamma \right)=p_{0}+\sum_{d=1}^{8}\left(p_{d}*\gamma^{d}\right)$

**Fig 6:**
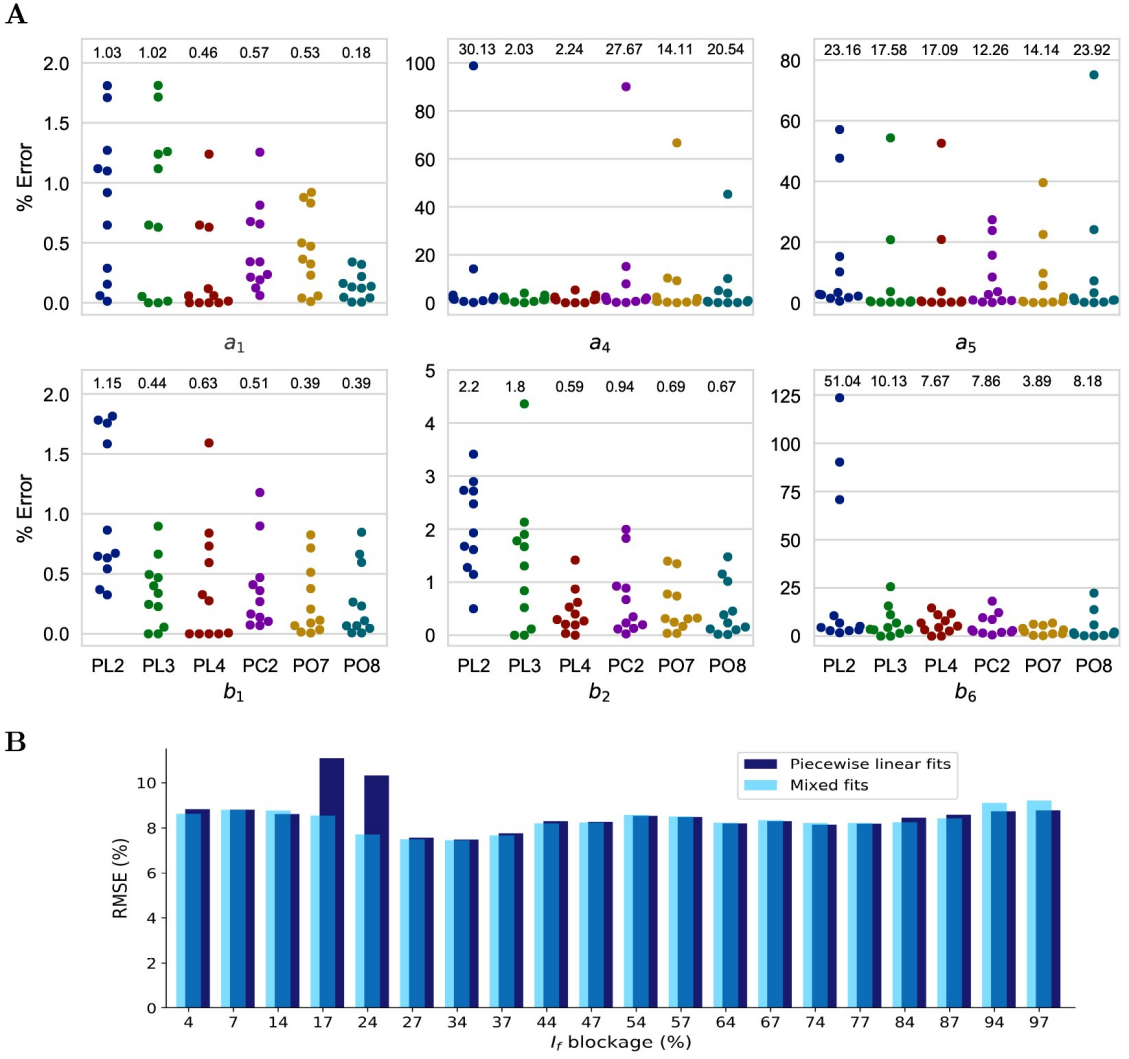
Selection of the small error and resource utilization friendly fitting curve to the coefficients for the Resonant model optimization. A: Percentage errors for different levels of *I*_*f*_ block and root mean squared errors (displayed at the top of every fit) for various types of fits to the resonant model coefficients. Piecewise linear with one breakpoint (PL2), piecewise linear with two breakpoints (PL3), piecewise linear with three breakpoints (PL4), piecewise cubic with one breakpoint (PC2), degree 7 polynomial (PO7), and degree 8 polynomial (PO8). B: Percentage root mean squared error (RMSE) for 20 *I*_*f*_ blockage percentage values when: 1) all the coefficients were fitted with minimum RMSE piecewise linear fits only (dark blue), 2) when all the coefficients were fitted with minimum RMSE fits, i.e., mixed fit types (light blue).

Fig 6A shows the percentage error, and root mean squared error (RMSE) for each of the six fits. The percentage error is the difference between the values of a RM coefficient (*K*_*ir*_) obtained by directly fitting the reference waveshape for the given current blockage and the coefficient (*K*_*if*_) evaluated using the equation of the corresponding fit-type. In Fig 6A, every fit-type for a RM coefficient has eleven % errors (represented as filled circles) corresponding to eleven different values of *γ*. The RMSE is calculated using Eq (10).

$$\begin{array}{c} \text{RMSE}=\sqrt{\frac{1}{11}\sum_{i=1}^{11}{\left(K_{ir}-K_{if}\right)}^{2}} \end{array}$$ (10)

In Fig 6A, the error subplot for *a*_1_ shows that the use of PO8 results in the smallest average RMSE of 0.18. In addition, all the eleven dots are closely clustered indicating an excellent overall fit. However, in the same subplot the coefficients obtained for *a*_1_ using PL4 show eight dots clustered around the zero error while three results in a higher error. Although there are twenty-two coefficients, only six have been selected to highlight our observations. The remaining eighteen coefficients are shown in S1 and S2 Figs.

***a_4_*** Here, PL3, i.e., three linear fitted equations results in the best fit with RMSE of 2.03. Also, all the eleven dots are closely clustered indicating an excellent overall fit.
***a_5_*** PC2 gives the smallest RMSE. However, the eleven dots are not closely clustered indicating a wider spread across the eleven AP waveshapes.
***b_1_*** Here, RMSE of 0.44 and visual interpretation show that PL3 closely follows both PO7 and PO8 which give the same minimum RMSE of 0.39. However, PL3 is computationally less expensive than PO7 and PO8.
***b_2_* and *b_6_*** PL4 and PO7 result in the best fit for *b*_2_ and *b*_6_, respectively.

These six subplots clearly show that a specific fit type is not the best fit for every coefficient. E.g., PO8 is suboptimal at all coefficients other than *a*_1_. However, the linear equations in PL2, PL3, and PL4 are computationally more attractive than the cubics and the polynomial equations of PC2, PO7, and PO8. The minimum error in AP waveshape is expected if the best fit type for each coefficient is individually selected. This best fit type for every coefficient is mentioned in Table 2 under the column heading ‘Mixed Fits’. The percentage RMSE for twenty interpolated values of *γ* is shown using light blue bars in Fig 6B. This error measures the deviation of the AP waveshape generated by RM when the coefficients are determined from the fitted equation and when the coefficients are obtained after fitting to reference AP waveshape. Since we intend to use linear fit types, we determined the best combination across PL2, PL3, and PL4 (Piecewise Linear Fits in Table 2). In Fig 6B, dark blue bars show the percentage RMSE using only linear fit types. The figure shows an insignificant difference between using a piecewise linear and mixed fits except for the funny current blockage levels of 17%-24%. This is the blockage range where the mixed fits perform slightly better than the piecewise linear fits. In addition, a Wilcoxon rank sum test was performed to test the null hypothesis that the AP waveshape data points obtained by using mixed fits and piecewise linear fits are from continuous distributions with equal medians. The p-values for twenty different values of *γ* vary from 0.0979 to 0.9907. This indicates the failure to reject the null hypothesis at the 5% significance level and conclude that the medians of the AP waveshapes data points from mixed fits and piecewise linear fits are equal. The Wilcoxon rank sum test results confirm the closeness between the mixed fits and piecewise linear fits.

**Table 2:** Fitting functions for the coefficients.

	Piecewise Linear Fits			Mixed Fits					
	PL2	PL3	PL4	PC2	PO7	PO8	PL2	PL3	PL4
**Coefficients**	-	*a*_4_ *b*_1_ *b*_7_	*a*_1_, *a*_2_ *a*_3_, *a*_5_ *a*_6_, *a*_7_ *a*_8_, *a*_9_ *a*_10_, *b*_2_ *b*_3_, *b*_4_ *b*_5_, *b*_6_ *b*_8_, *b*_9_ *a*_0_, *ω*	*a*_5_	*a*_1_ *a*_2_ *a*_8_ *a*_9_	*a*_3_, *a*_4_ *a*_6_, *a*_7_ *b*_2_, *b*_3_ *b*_4_, *b*_5_ *b*_7_, *b*_9_ *b*_10_, *a*_0_ *ω*	-	*a*_4_	*a*_10_ *b*_6_

### Resonant model development for cardiac myocyte

**Fig 7:**
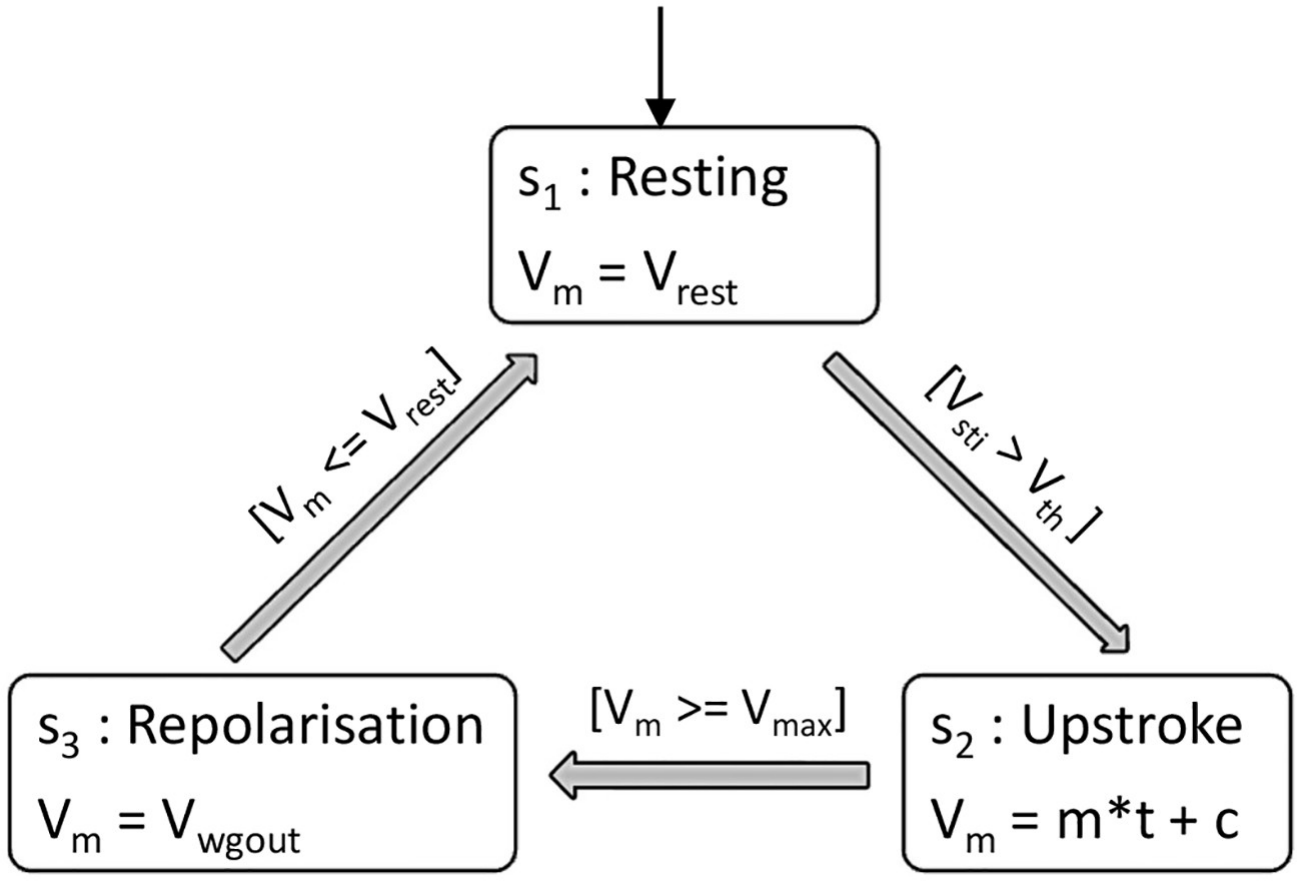
State controller of Resonant model for ventricular myocyte. V_m_ = membrane potential, V_rest_ = resting membrane potential, V_sti_ = voltage stimulus to a cell, V_th_ = threshold membrane potential, V_max_ = maximum membrane potential, and V_wgout_ = membrane potential from waveshape generator.

Recall that the self-excitatory property together with mutual entrainment requires a higher layer of decision and control of the oscillators. However, in the case of myocytes the absence of inherent auto-rhythmicity, the decision and control layer needs to be modified. The modified state controller that implements a myocyte centric decision and control is shown in Fig 7. We implemented the state controller using a state flowchart in SIMULINK. The states s_1_, s_2_ and s_3_ model the dynamics of the AP in the resting, upstroke and repolarization phase, respectively. The membrane potential is defined by a piecewise-continuous variable V_m_. V_sti_ represents the stimulus required to excite the cell and has contributions from external sources like drugs or neighboring myocytes. In steady state, the myocyte is at its resting potential V_rest_ in state s_1_. The transition from s_1_ to s_2_ phase is triggered when the invariant condition V_sti_ > V_th_ becomes true, where V_th_ is the threshold membrane potential. In this state the V_m_ depolarizes. This state has been modeled with an equation of a straight line. After the cell reaches maximum potential (V_max_), it goes through the repolarization phase, and the non-linear V_m_ is produced by waveshape generator of the resonant model. After repolarisation, the cell reaches its resting potential where it remains until the arrival of an appropriate stimulus. The oscillators are only active in state s_3_.

### Action potential parameters

The AP of cell types were characterized by various action potential parameters. For SAN cell type, the maximum diastolic potential (MDP) is the most negative value of membrane potential. Action potential amplitude (APA) is the amplitude of the membrane potential, measured as a difference between the positive peak potential and the maximum diastolic potential. Action potential duration at an arbitrary repolarization potential of X% (APD_X_) is the time between 50% depolarisation and X% repolarization of the action potential. Overshoot (OS) is the maximum positive potential. Diastolic depolarization rate during a 100 ms time interval (DDR_100_) is a change in membrane potential in 100 ms starting at a membrane potential that is 1 mV positive to the MDP. Interbeat interval (IBI) is the time between 50% depolarisation on the upstroke of a beat and 50% depolarisation on the upstroke of next beat. For a cardiac myocyte, we determined APD at X% of complete repolarization as the time between the start of the AP and when membrane voltage had returned to X% of the resting value. The resting potential was measured prior to the onset of the beat. The start of the AP was the time at which maximum change in membrane potential occurred. However, for a RM cell, it was taken as the time when the cell started to depolarize as the upstroke was modeled as a straight line.

### Numerical implementation and simulations

The resonant models of a human and rabbit SAN cell, cardiac myocyte, and a neuron were built in SIMULINK version 6.4 (The Mathworks, Inc.). Simulations ran on a Windows 7 PC equipped with an Intel Core i7 3.4 GHz processor. Numerical integration was performed by ode 15s. Numerical results were visualized using MATLAB R2016b (The Mathworks, Inc.). Simulations of detailed electrophysiology models were run until steady-state was reached. An approximate steady state was taken as the condition in which no further change in the cell’s cycle length was observed.

## Results

### Autorhythmic cells

#### Action potential morphology

**Fig 8:**
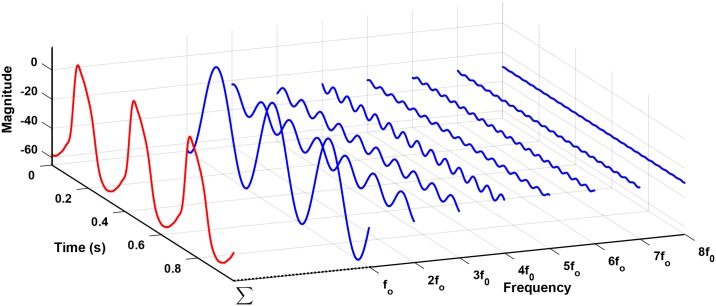
The contribution of fundamental and seven harmonics to produce an autorhythmic cell AP. AP waveshape (red) is the result of the superposition of fundamental and seven higher frequency components (blue) of Fourier series.

A part of Fig 2B has been redrawn in Fig 8 to highlight the selection of the number of harmonics. The figure shows the AP generated by the RM with eight oscillators. The output of every oscillator is shown separately corresponding to their frequency of oscillation. The fundamental frequency is *f*_0_. The AP waveshape (red) results from the superposition of all the outputs of the oscillators and a dc offset. The higher the harmonic, the lesser is its impact on the output AP waveshape. Fig 8 highlights that the impact of the 8th harmonic is minimal in comparison to the magnitude of the fundamental frequency component. Therefore, AP waveshape can be represented with the RM comprising a finite number of oscillators. The adjusted R squared value of this AP waveshape generated by RM with eight oscillators is 0.9929, which means that it captured more than 99% of the variability of the reference AP waveshape data.

**Fig 9:**
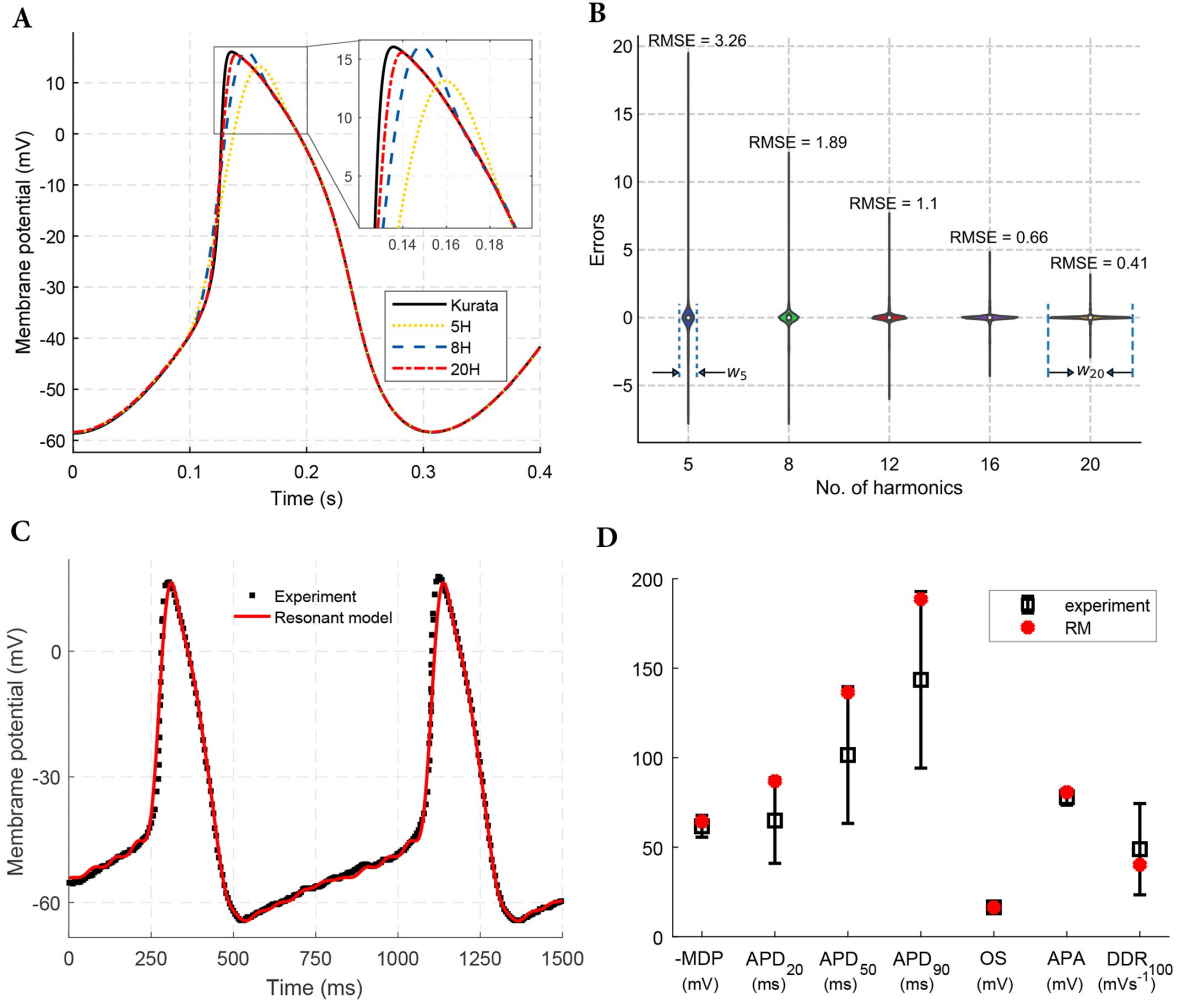
AP morphologies of autorhythmic cells. A: Comparison of rabbit SAN cell AP morphology (black solid) from Kurata model and AP morphologies obtained from RM with 5 (green dotted), 8 (blue dashed), and 20 (red two dashes) harmonics. B: Violin plots of errors with the variation in the no. of harmonics in the finite Fourier series for reproducing a rabbit SAN cell AP. Also shown is the root mean squared error corresponding to each harmonic. *w*_5_ and *w*_20_ indicates the maximum thickness of the violin plot of errors for 5 and 20 partial sums, respectively. C: RM (12 harmonics) fit to experimental human sinoatrial node cell’s AP. Note: The experimental data has been regenerated from Fig 5A in [33] D: Comparison between experimental and simulated AP features of human SAN cell. Here, MDP is Maximum diastolic potential; APD_20_, APD_50_, and APD_90_ are action potential durations at 20, 50 and 90% repolarization, respectively; OS is the overshoot; APA is the action potential amplitude; DDR_100_ is the diastolic depolarization rate over the 100 ms time interval starting at MDP.

Fig 9A shows the AP morphologies of rabbit SAN cell obtained from the Kurata et al. model [22] compared with the output of various RMs. A close inspection reveals that with the increase in the number of harmonics, the accuracy of the RM also increases. The parameters for the RM with different number of harmonics are listed in S1 Table. The violin plot in (Fig 9B) shows the inverse dependence of errors on the number of harmonics taken into account. For example, with only five harmonics there are few samples with a large error. However, with twenty harmonics there are few samples with maximum error reduces significantly. Also, with twenty harmonics there are many more samples with small errors. The error range decreases and the width of the violin plot, corresponding to zero error, increases with an increasing number of harmonics, *w*_20_ > *w*_5_, as shown in the figure. We quantified the accuracy of the fits with various harmonics by determining the root mean squared error between the outputs of the Kurata et al. model [22] and our RM (Fig 9B). However, more oscillators in the RM implies an increase in the computational load and/or resource utilization. Thus, there is a tradeoff between fidelity and execution time and is discussed in detail in Discussions.

To further validate the capability of RM to reproduce autorhythmic cell’s AP waveshape, the experimentally recorded AP of single human SAN autorhythmic cell (adapted from [33]) was reproduced with RM consisting of twelve oscillators (Fig 9C). The parameters for the RM for human SAN are mentioned in S2 Table. Indeed, most of the quantitative parameters that describe the AP morphology are within one standard deviation of the means of the experimental values [34]. In Fig 9D, the qualifying metrics of an APD are clearly well aligned with human SAN cell measurements. The human SAN cell AP generated by the RM is characterized by a cycle length (CL) of 827.9 ms, corresponding to a beating rate of 72 beats min^-1^. However, the only metric beyond the empirical measurements is the (d*V*/d*t*)_max_. The RM results in a lower (d*V*/d*t*)_max_. This is inevitable because the highest component in the RM is 12th harmonic. However, if a requirement imposes this metric to be achieved, then higher harmonics can be included. It should be noted that in the electrophysiological model [34], three of these quantifying metrics are not within the standard deviation of the experimental values. An implementation criterion to meet a design requirement will rationalize the number of oscillators in the RM.

#### Coupled SAN cell pair synchronization

**Fig 10:**
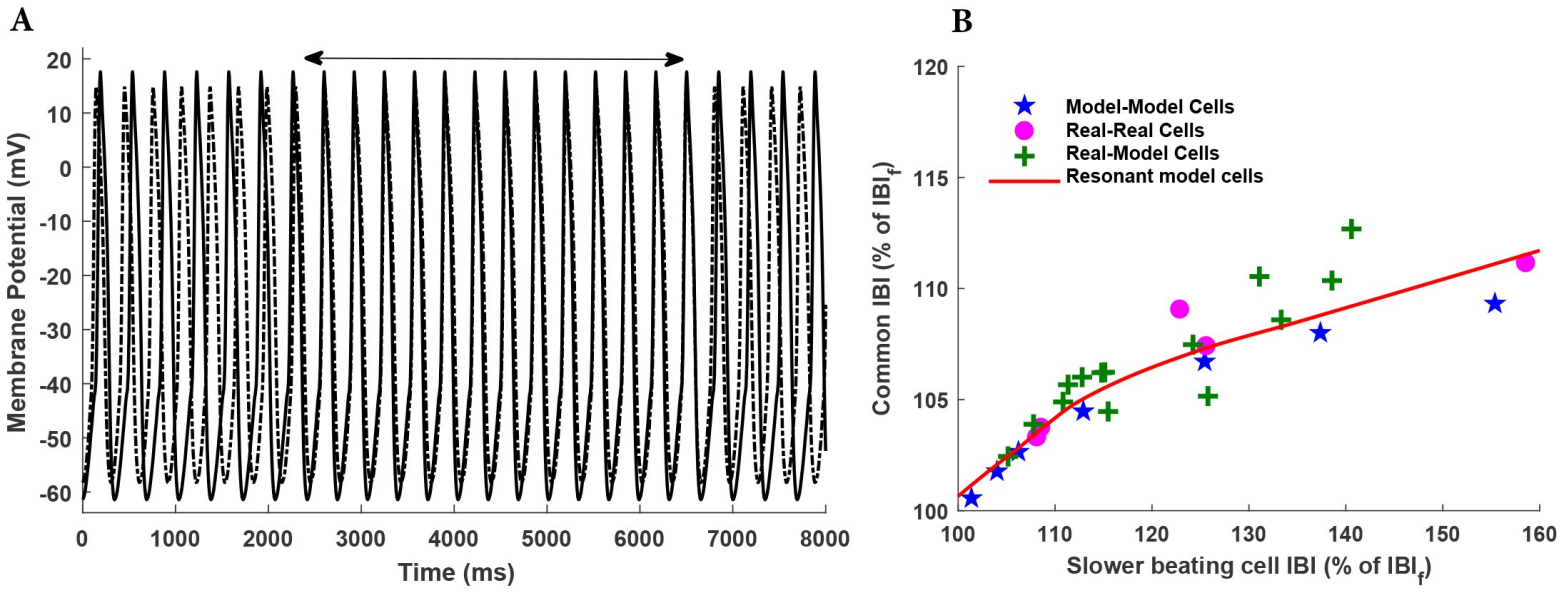
Mutual entrainment of SAN cells. A: Simultaneous recordings of membrane potential of two rabbit SAN Resonant model cells, with the cells coupled for the period indicated by the horizontal two-headed arrow. B: Common interbeat interval (IBI) (as a percentage of the IBI of the intrinsically faster beating cell, IBI_f_) versus the IBI of the slower beating cell of the coupled cell pair (also as a percentage of the IBI_f_). Results obtained from a simulation of two Kurata et al. model pacing cells are represented by blue stars [32]. Magenta circles show the experimental results of isolated real rabbit SAN cell pair; green plus represents the results from coupling of real rabbit SAN cell with the model cell [35], and the solid red line shows the common IBI for resonant model SAN cell pair.

RM rabbit SAN cells (8 oscillators) with different cycle lengths were coupled. The variation in the intrinsic cycle length (CL) was achieved by reducing the sustainability current (*I*_*st*_) by a fractional amount in Kurata et al. model [22]. To achieve this, the maximal conductance (*g*_*st*_) was reduced by the fraction 8/19. This reduction results in CL disparity of approx. 12%–56%, which is in the range of experimental values of CL disparity of 10%–60% [32]. The RM coefficients were obtained for all the different beating frequencies of AP waveshapes. The RM SAN cells were coupled through the gap junction represented by Eq (8). Fig 10A shows simultaneous simulated outputs from two RM rabbit SAN cells, with the membrane potential recordings of two cells distinguished by a solid (for cell A) and dash-dotted (for cell B) line. The cells A and B were uncoupled for 2400 ms, followed by a period of 4000 ms of electrical coupling, and then by the second period of uncoupling of 1600 ms. During the period of uncoupling, the spontaneous activity of cell A was occurring at a longer interbeat interval (345.8 ms) than the spontaneous activity of cell B (306.8 ms). The AP waveshapes of the two cells were also different, with cell A having a more negative maximum diastolic potential (-61.4 vs. -58.3 mV) and a more positive peak amplitude (17.6 vs. 14.9) than cell B. During the 4 s of coupling in Fig 10A (as indicated by the horizontal two-headed arrow), the AP waveshapes of cells A and B were synchronised to each other in a 1:1 manner with the common IBI of 325 ms and exhibited mutual entrainment. This common IBI is biased towards the faster cell. Fig 10B plots the common IBI as a function of the intrinsic IBI of the slower cell. Note that the common IBI and the slower IBI (IBI_s_) are expressed as percentages of the intrinsic IBI of the faster cell, IBI_f_. The traces show that the entrainment as a result of coupling two RM SAN cells (solid red line) closely matches the entrainment of the two biological SAN cells (magenta circles). However, the coupling of two detailed electrophysiology model cells (Kurata et al. model) (blue stars) shows a deviation at lower beating frequencies. S4 Fig shows simultaneous simulated outputs from 1D network consisting of five rabbit SAN cells. All the five cells have different intrinsic beating frequencies as shown in the uncoupled region. During the period of coupling (as indicated by the arrow), the AP waveshapes of all the cells were synchronized and exhibited mutual entrainment.

#### Current blockage effects

**Fig 11:**
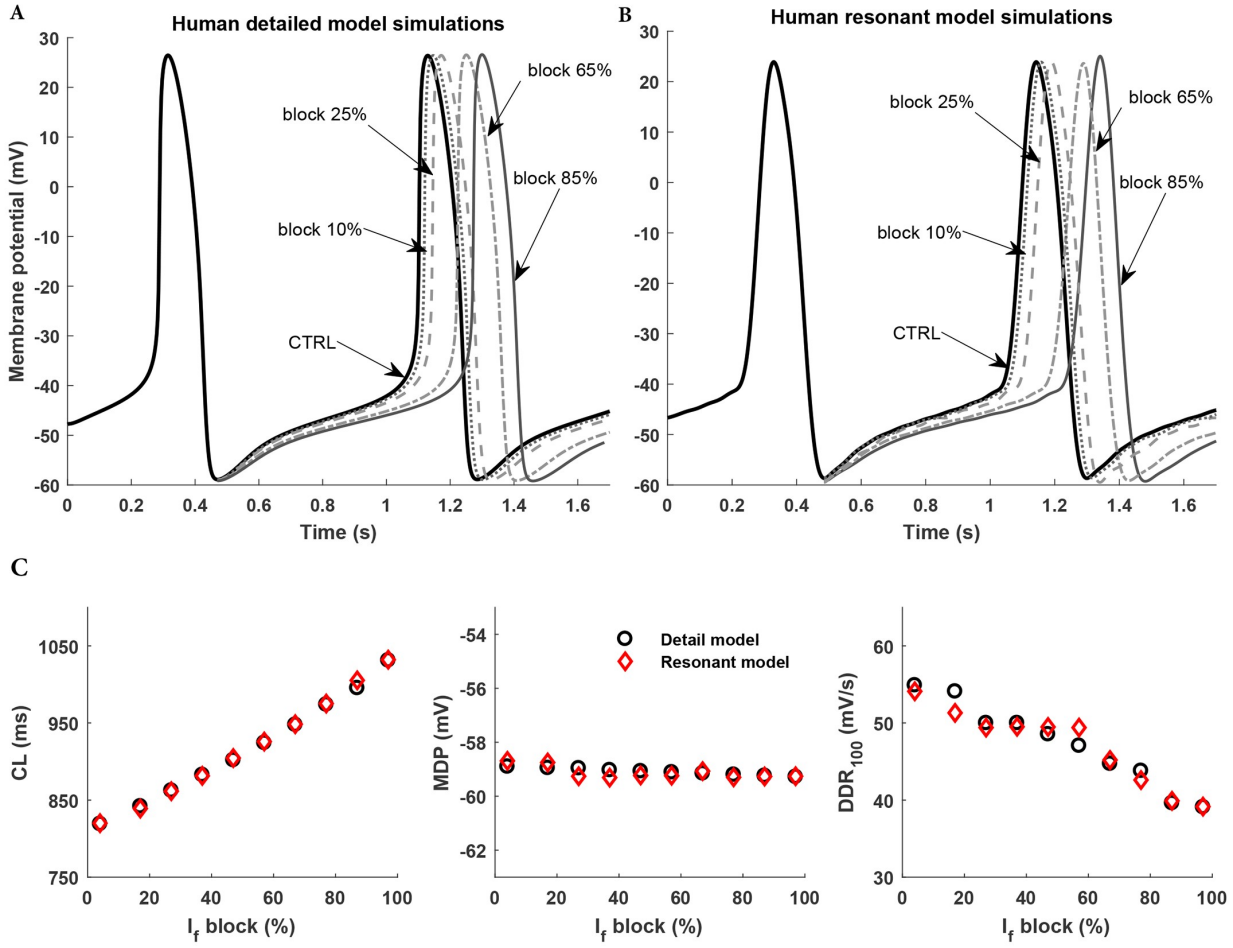
Human sinoatrial node detailed electrophysiology model and human sinoatrial node Resonant model responses to perturbation in an ion transport pathway. A: Action potential waveforms simulated in human sinoatrial node cell electrophysiology model. B: Action potential waveforms simulated in human sinoatrial resonant model under control conditions (CTRL) (dark black solid line) and upon 10% (dotted line), 25% (dashed line), 65% (dash-dot line), and 85% (black solid line) block of *I*_*f*_. C: Simulated effects of different levels of *I*_*f*_ blockage on cycle length (CL), maximum diastolic potential (MDP) and diastolic depolarization rate (DDR_100_).

We developed a methodology which makes the RM capable of producing the electrophysiological responses to drugs. The quantitative effect of the funny current (*I*_*f*_) on the cycle length (CL), and thus to the pacing rate, of the human SAN cell has been examined using the Fabbri et al. model [21]. The detailed model was simulated at eleven different maximal conductances *g*_*f*_ to determine the prediction model. Fig 11A and 11B show the simulations of the *I*_*f*_ blockage using the Fabbri et al. model and the predictions of the RM, respectively. Although, these figures highlight the AP comparison at 10%, 25%, 65% and 85%, Fig 11C draws out a comparison of other predicted AP waveshape metrics: CL, MDP and DDR_100_. Both CL and MDP closely tracks the electrophysiology model. Although, DDR_100_ exhibits error of approximately 3%, in most instances the RM closely tracks the Fabbri et al. model. The quantitative effect of the total block of *I*_*f*_ in the RM led to an increase in CL of 27.9% to 1.04 s, and thus an approximate 22% decrease in pacemaking rate to 58 beats min^-1^ (approx). These measurements were identical in Fabbri et al. model. The results were also in good accordance with the 26% increase in the CL that was experimentally observed by Verkerk et al. [34]. In his experiment with a single isolated human SAN cell, *I*_*f*_ was almost completely blocked by the administration of a 2mM Cs^+^. Of note, the increase in CL was largely the result of a longer diastolic depolarization phase, whereas APD_90_ and MDP almost remained unaffected in the experiment done by Verkerk et al. The similar results were produced by the parameterized RM of human SAN cell (Fig 11C) as well. DDR_100_ was the main descriptive parameter that decreased by 26.6% to 38.97 mVs^-1^ in Fabbri et al. model and by 27.2% to 39.07 mVs^-1^ in the resonant model of human SAN cell.

### The Resonant model approach is generalizable for multiple cell types

**Fig 12:**
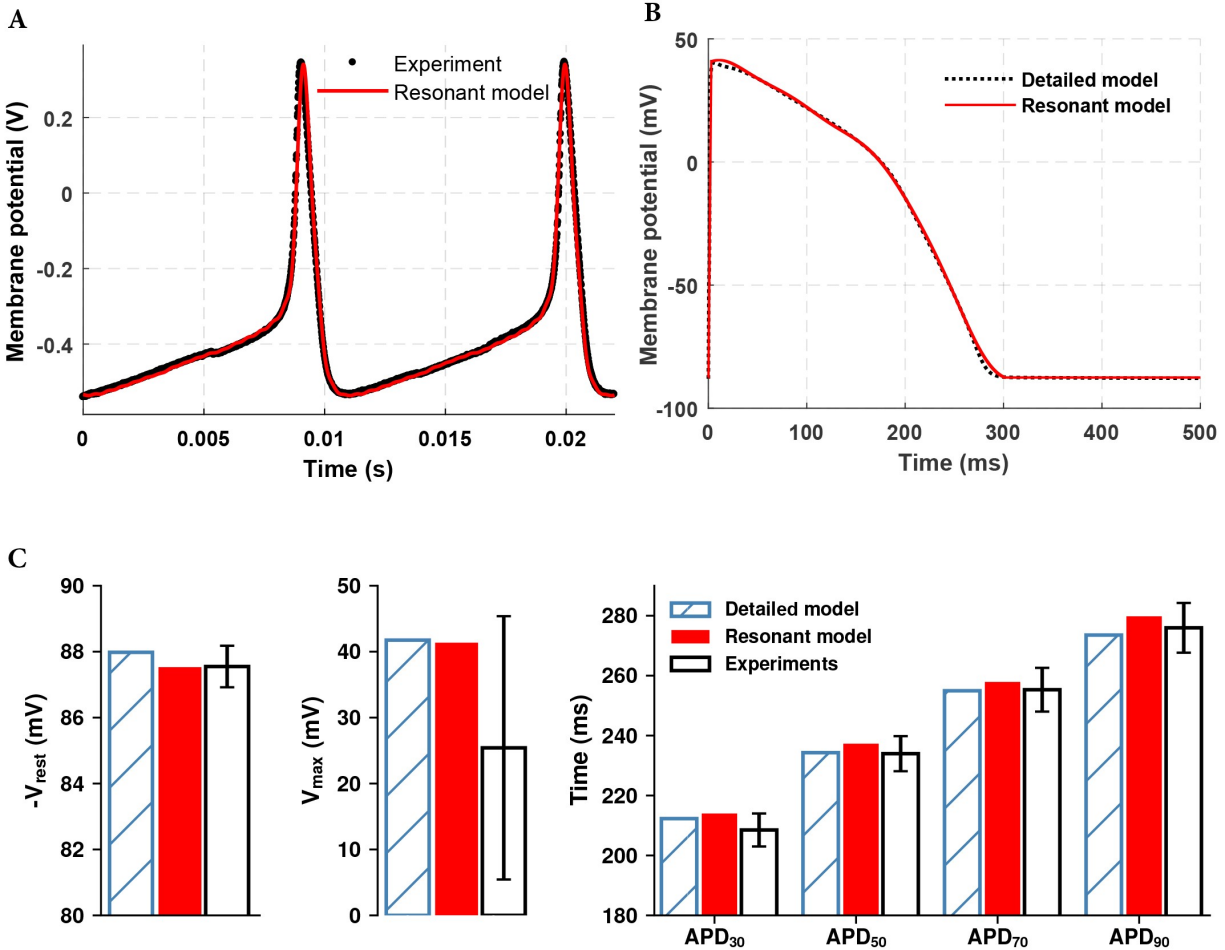
Extension of the Resonant model to additional cell types. A: Simulated resonant model AP morphology (solid red line) in comparison with experimental trace (black dots) of squid giant axon membrane potential. B: Human ventricular myocyte APs generated by the simulation of resonant model (red solid line) and O’Hara et al. model (black dashed line) C: Comparison of simulated O’Hara et. al (blue hatched), resonant model (red) and experimentally measured (black) basic AP parameters of human ventricular myocyte for a single paced beat from quiescence. Shown, from left to right, are the negative resting membrane potential (V_rest_), maximum upstroke potential (V_max_), and action potential duration at 30, 50, 70, and 90% repolarization.

We have developed RM models for a neuronal cell and a cardiac myocyte. The neuronal AP was obtained experimentally by Paydarfar et al. [36, 37]. As compared to the human SAN AP waveshape, the electrical response of a neuron is swift and sharp. This necessitates the inclusion of more oscillators in the RM to improve the closeness of the fit. Fig 12A shows the simulated RM (20 oscillators) AP waveform proving that the RM is capable of generating challenging waveshapes.

Fig 12B shows the simulated AP morphology generated from the O’Hara et al. electrophysiological model of the human endocardial myocyte [20] at the pacing cycle length of 1000 ms. Also shown in this figure is the RM model’s equivalent simulation. Microelectrode AP recordings from undiseased human ventricular endocardium were used to validate primary RM AP characteristics [20]. Comparison of RM simulated values for resting voltage V_rest_, maximum upstroke voltage V_max_, action potential duration at 30, 50, 70, and 90% repolarization, with corresponding experimentally obtained values and O’Hara et al. model simulated values are shown in Fig 12C. RM for human ventricular cells quantitatively reproduced the AP morphology as the RM simulation results were within experimental error bars and very close to results obtained from O’Hara et al. model simulations.

## Discussion

In this paper, we have proposed a new modeling technique called as a Resonant model (RM) to describe the electrical behavior of biological cells observed in vivo or in vitro via simplified in silico representation of their action potential morphology. The model predicts experimental properties and characteristics of biological cells, including (1) human and rabbit SAN AP morphology, (2) current blockage effects, (3) frequency entrainment in autorhythmic cells, (4) human cardiac myocyte AP morphology, and (5) squid giant neuron AP morphology. We have validated the AP morphology produced by the RM of the human autorhythmic cell and human cardiac myocyte. Also, the frequency entrainment exhibited by the RM of human SAN has been validated against experimental results. The two components of the RM are the waveshape generator and the state controller. The Waveshape generator produces basic AP waveshape and consists of oscillators. Each oscillator represents a harmonic in the Fourier series. RM is capable of producing very different and complex AP waveshapes with the same modeling framework as we have seen in case of SAN cell and neuron. Both cells possess markedly different AP characteristics with a neuron firing very fast with sharp spike compared to the slow paced SAN cell. However, RM captured these AP morphologies by just varying the number of oscillators. This adds computational certainty since the numerical behavior of each oscillator will be the same. To incorporate various dynamic properties of a cell, state controller was designed with the help of a stateflow^®^ that includes various functions. These functions are evaluated only once on the change of any working condition and this results in a minimal increase in the computational cost. The results (Fig 9) show that that with the increase in the number of harmonics, the accuracy of the RM also increases. However, more oscillators will result in an increase in execution time, and thus there is a tradeoff between fidelity and execution time. Also, the parametric variation of AP morphology could be used to model the behavior of new drugs. Administering of a new drug may lead to the blockages of one or more ionic currents. With respect to *γ* in Eq (4). *γ* could be proportional to the concentration of the new drug. Although the drug could influence multiple ionic channels, the impact on the waveshape morphology can be parametrized by a single variable (*γ*).

For comparison, we quantitatively analyzed the complexity of detailed and generic electrophysiology models with respect to the RM. The results are presented in Table 3. The results show the average number of data points computed per second by the Central Processing Unit (CPU) for different models. All the models were simulated in discrete time in MATLAB at various time steps eighty times for 5000 ms. The detailed electrophysiology models [21, 22] with more than 27 state variables are twenty-five times complex than the RM with twelve Fourier terms (FT). Also, the detailed electrophysiology models require the use of smaller step size in the numerical computations compared to the RM. We recognize the fact that the detailed electrophysiology models are capable of capturing complex behaviors. However, for specific applications such as tissue simulations at large scale involving millions of grid points, an alternative technique that results in high speedups is requisite. The results indicate that the RM is twice as fast as the simplest three-variable model of the cardiac action potential [38]. This comparison shows that RM improves the computational tractability while accurately reproducing most of the action potential characteristics.

**Table 3:** Computing load of the different computational biological cell models.

Model Name	Type of a biological cell	No. of variables	No. of constants	No. of data points/s (x 10^5^)
Fabbri et al. model [21]	human sinoatrial node	33	116	3.26
Resonant model (12FT)	human sinoatrial node	24	26	185.44
Kurata et al. model [22]	rabbit sinoatrial node	27	66	4.81
Resonant model (8FT)	rabbit sinoatrial node	16	18	340.28
Fenton FH, Karma A. model [38]	cardiac cell	3	22	130.06
Resonant model (4 FT)	human ventricular cell	8	16	265.27

If graphic processing unit (GPU) and field programmable gate array (FPGA) are being considered as implementation platforms, the potential parallelism offers significant improvements in fidelity without compromising execution times. RM is tractable to both time-division multiplexing (TDM) and parallelism. Given that the beat frequencies of the biological cells are extremely low compared to CPU clock frequencies, several oscillators can be updated within one sampling instant. The time-division multiplexing would heavily rely on fast computing of sine/cosine function. Hardware trigonometric calculations are a common place on computers for several decades. Each harmonic will require two additions and one cosine function call. A RM framework can be implemented as a discrete time kernel of two integrators in a closed loop with negative feedback. This implementation requires only two additions and two shift operations. This is possible because the initialization of each kernel circumvents the need to accommodate scaling and phase shifting of each harmonic.

On FPGAs, both TDM and parallel frameworks are possible. The two most popular techniques for trigonometric calculations are a CORDIC algorithm and a lookup-table. Either of these two techniques can be selected to strike a balance between accuracy and resource usage. The RM strategy, when implemented, eventuates as a discrete-time kernel for each oscillator. The multiplier required in the Fourier series can be eliminated by appropriately initializing each integrator. In addition, both TDM or parallel implementation techniques can be optimized based on the implementation platform. Thus, the implementation of the RM offers resource usage advantages in both TDM and parallel implementations. The RM framework is also amenable to parallel hardware implementation because every oscillator is independent of other oscillators. This schematic could be realized on FPGA by using only two additions whereas CORDIC and lookup table in comparison utilizes many more resources. Table 4 shows the different operations required to get one cosine value. Total number of fixed-point operations in different techniques depend on the number of samples required to generate one beat with a specific accuracy.

**Table 4:** Cost comparison of the fixed-point approximation algorithms for a sinusoid calculation.

Fixed-point CORDIC	Single lookup table	Resonant model oscillator
3 additions per iteration	1 addition	2 additions
2 shifts per iteration	1 multiplication	2 shifts
1 table lookup per iteration	1 table lookup	-

Although the results presented in this study have demonstrated that the developed RM quantitatively reproduced AP morphologies of various biological cells and provided confidence that the concept can be of practical use, several limitations should be noted. First, there can be multiple sets of coefficients of RM with the same goodness of fit due to the existence of more than one local minima. To overcome this, statistical fitting algorithms capable of finding global minima like genetic algorithms [39, 40], simulated annealing and particle swarm optimization could be used. Second, depending upon the goodness of fit, sometimes ripples are present in the AP waveshapes generated by the RM. However, in most of the cases, the magnitude of the ripples is smaller than the noise magnitude in the experimental recordings. Although each step in model parameter determination can be automated, human oversight is required to ensure correct algorithmic decisions are made (e.g., breakpoint determination in Fig 5).

Although the four different cells modeled in this study provide a stong proof-of-concept for the modeling approach, to further develop the method, our RM methodology can also be applied to other biological cells of various species like atrial myocytes, atrioventricular nodal cells, skeletal muscle cells or plant cells [41]. More dynamic properties possessed by biological cells, like action potential duration rate dependence and action potential duration restitution can also be included. In addition, by integrating a geometric model with suitable models of the AP in single cells, it is possible to reconstruct the electrical activity in: 1D fibre to address critical aspects of the origin and the spread of cardiac excitation; a 2D model to study complex behaviors like dysrhythmias. The RM adds a new low computational cost modeling approach to the family of modeling techniques developed in previous studies for various biological cells, which can be incorporated into an anatomical model of the human organ with details of its structural uniformity and anatomical complexity. Fully optimized real-time implementation of RM can also be further investigated.

## Conclusion

We have developed a novel mathematical model for various types of biological cells that serves to capture the electrical activity of a cell with quantitative accuracy. Our resonant model can be applied to various cell types as well as to different species. It can generate accurate action potential morphologies as well as respond to current blockage effects and also demonstrates frequency entrainment. Our model accurately matches clinical data and the simulated dynamics of detailed electrophysiology models. Furthermore, the speedup of the resonant model will narrow the gap between the simulation/emulation time and the real-time requirement for tissue level simulations. The resonant model development methodology can easily be applied to other fields in which processes are periodic or quasi-periodic, potentially significantly increasing the impact of real-time emulations.

## Supporting information

S1 Fig Percentage errors for different levels of *I*_*f*_ blockage and root mean squared errors (displayed at the top of every fit) for various types of fits to the Resonant model coefficients. Piecewise linear with one breakpoint (PL2), piecewise linear with two breakpoints (PL3), piecewise linear with three breakpoints (PL4), piecewise cubic with one breakpoint (PC2), degree 7 polynomial (PO7), and degree 8 polynomial (PO8).
(PDF)

S2 Fig Percentage errors for different levels of *I*_*f*_ blockage and root mean squared errors (displayed at the top of every fit) for various types of fits to the Resonant model coefficients. Piecewise linear with one breakpoint (PL2), piecewise linear with two breakpoints (PL3), piecewise linear with three breakpoints (PL4), piecewise cubic with one breakpoint (PC2), degree 7 polynomial (PO7), and degree 8 polynomial (PO8).
(PDF)

S3 Fig Minimum root mean squared error fits to the coefficients of the Resonant model (RM) of a human sinoatrial node cell at different levels of *I*_*f*_ blockage. The black diamonds represent the RM coefficients obtained after fitting to AP waveshapes of Fabbri et al. model for specific percentage values of *I*_*f*_ blockage. Dotted lines represent the fits to the coefficients. The single color fit (red) is either 7 degree or 8 degree polynomial. Fits to coefficient values with different colors are either piecewise linear or piecewise cubic. Coefficients of every oscillator in the RM are placed together in a black rectangular box. *ω* represents the fundamental frequency.
(PDF)

S4 Fig Mutual entrainment in 1D network of SAN cells. Simultaneous recordings of five rabbit SAN Resonant model cells, with the cells uncoupled and coupled as indicated by the arrow.
(PDF)

S1 Table Resonant model coefficient values for generating rabbit SAN AP. (PDF)

S2 Table Resonant model (12 oscillators) coefficient values for generating human SAN AP. (PDF)
