# Supplementary figures and images for: Resonant model—A new paradigm for modeling an action potential of biological cells
Source: PLoS One. 2019 May 22;14(5):e0216999. doi: 10.1371/journal.pone.0216999 (PMC6530846; doi:10.1371/journal.pone.0216999)

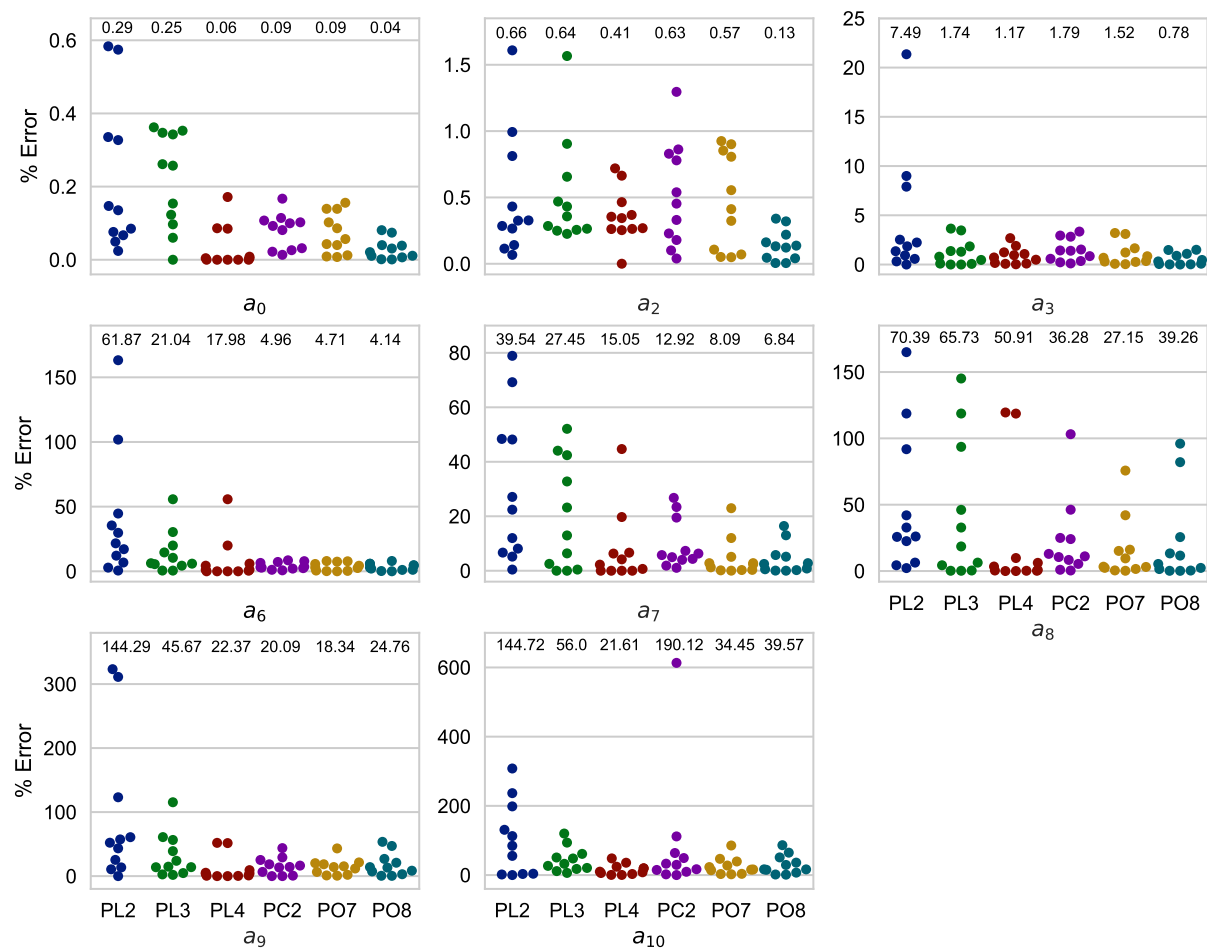

Supplement: S1 Fig — Piecewise linear with one breakpoint (PL2), piecewise linear with two breakpoints (PL3), piecewise linear with three breakpoints (PL4), piecewise cubic with one breakpoint (PC2), degree 7 polynomial (PO7), and degree 8 polynomial (PO8). (PDF) [file pone.0216999.s001.pdf]

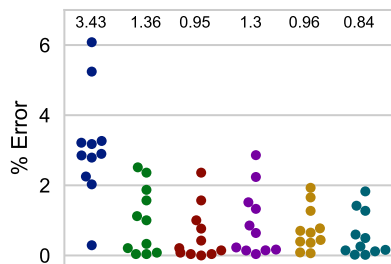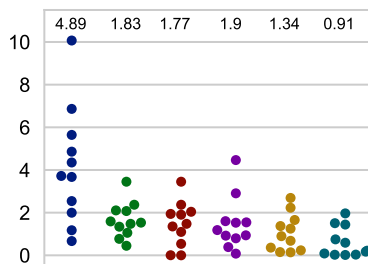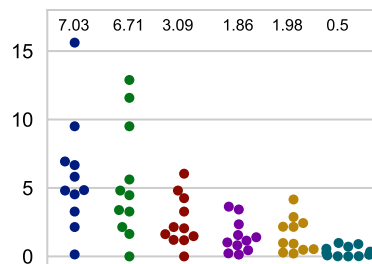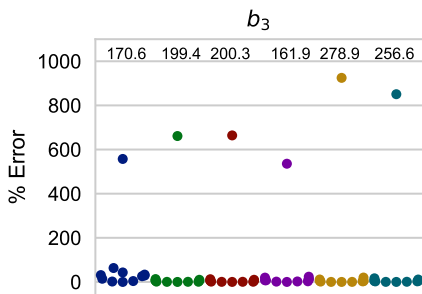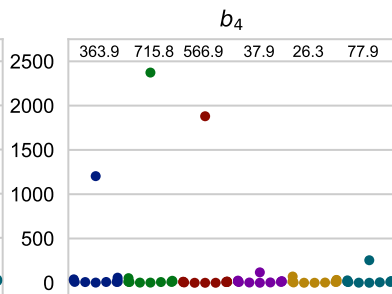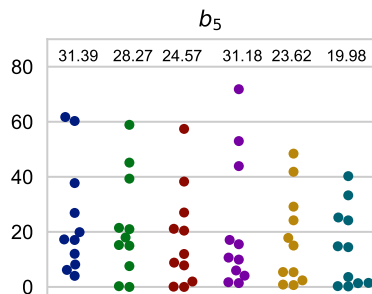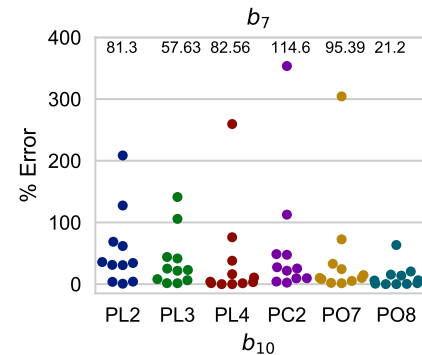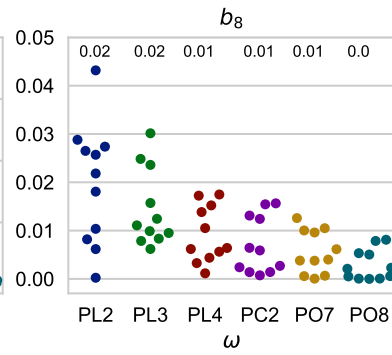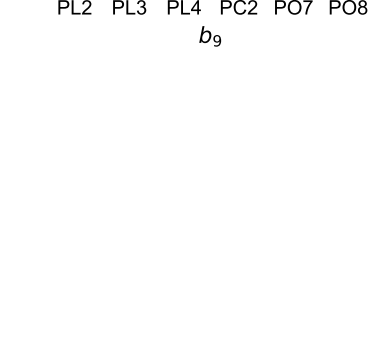

Supplement: S2 Fig — Piecewise linear with one breakpoint (PL2), piecewise linear with two breakpoints (PL3), piecewise linear with three breakpoints (PL4), piecewise cubic with one breakpoint (PC2), degree 7 polynomial (PO7), and degree 8 polynomial (PO8). (PDF) [file pone.0216999.s002.pdf]

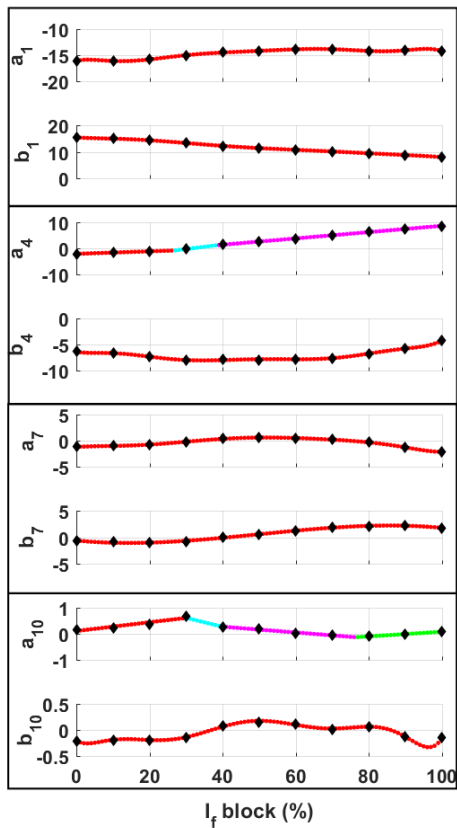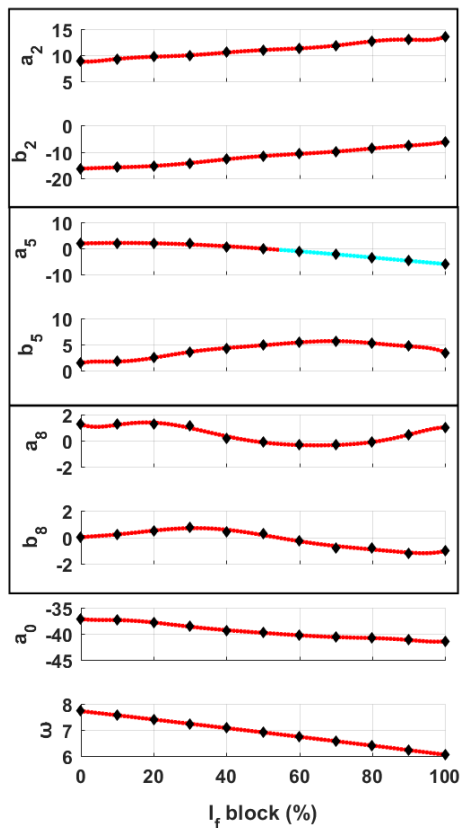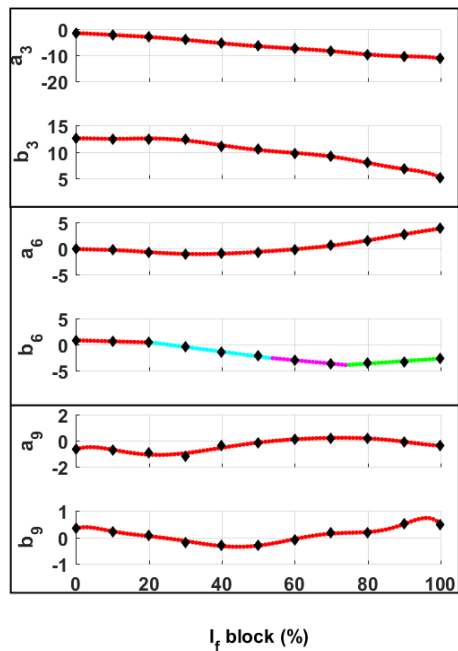

Supplement: S3 Fig — The black diamonds represent the RM coefficients obtained after fitting to AP waveshapes of Fabbri et al. model for specific percentage values of If blockage. Dotted lines represent the fits to the coefficients. The single color fit (red) is either 7 degree or 8 degree polynomial. Fits to coefficient values with different colors are either piecewise linear or piecewise cubic. Coefficients of every oscillator in the RM are placed together in a black rectangular box. ω represents the fundamental frequency. (PDF) [file pone.0216999.s003.pdf]

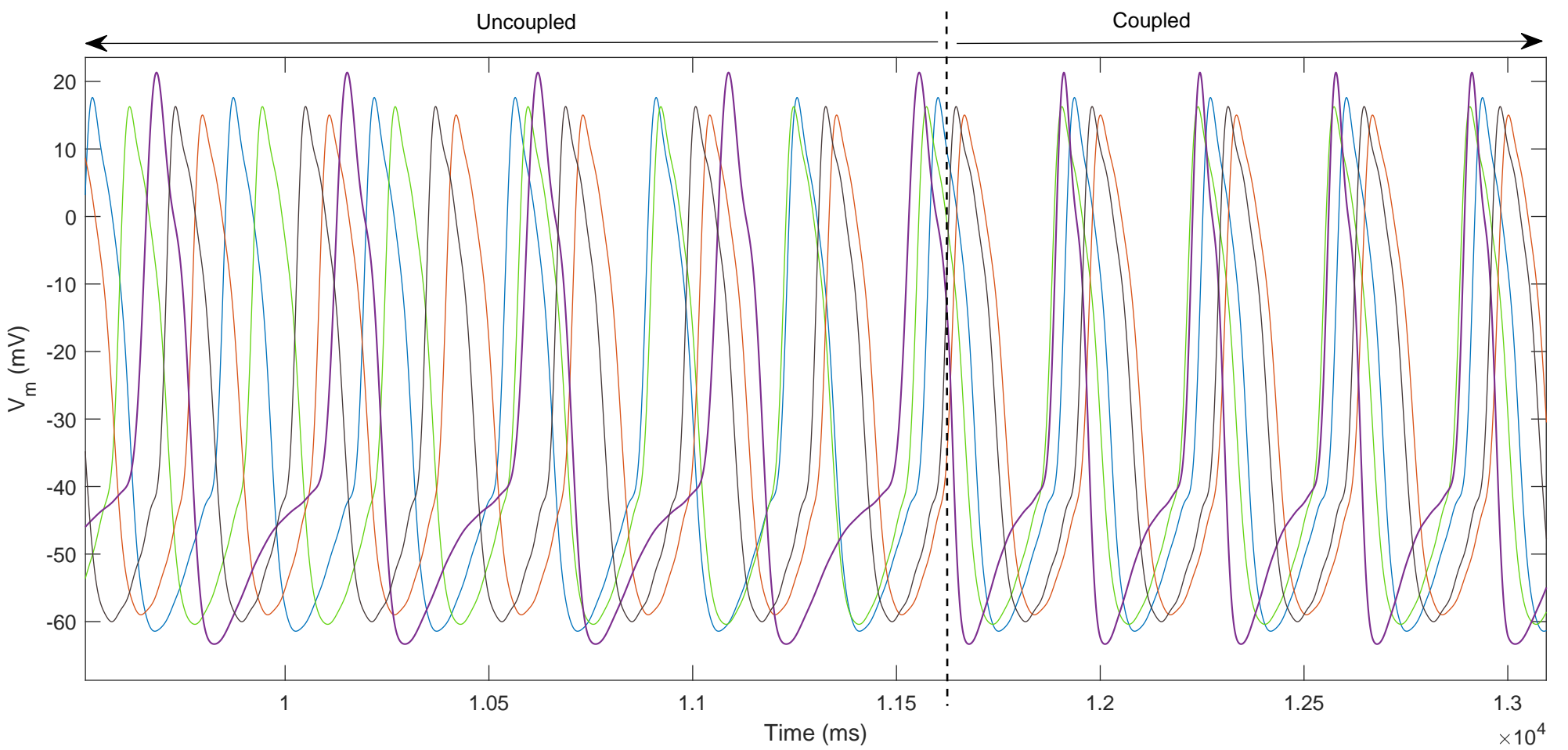

Supplement: S4 Fig — Simultaneous recordings of five rabbit SAN Resonant model cells, with the cells uncoupled and coupled as indicated by the arrow. (PDF) [file pone.0216999.s004.pdf]
