# Supplementary material for: Resonant model—A new paradigm for modeling an action potential of biological cells
Source: PLoS One. 2019 May 22;14(5):e0216999. doi: 10.1371/journal.pone.0216999 (PMC6530846; doi:10.1371/journal.pone.0216999)
Supplement: S1 Table — (PDF) [file pone.0216999.s005.pdf]

S1 Table. Resonant model coefficient values for generating rabbit SAN AP

Sub system	5 Subsystems				8 Subsystems				12 Subsystems			
	Integ1 IC*	Integ IC	freq	a_0	Integ1 IC	Integ IC	freq	a_0	Integ1 IC	Integ IC	freq	a_0
**1**	-31.93	11.2	20.46	-31.21	-33.16	10.650	20.46	-30.57	-33.16	10.51	20.46	-30.54
**2**	5.734	-6.062	40.92		6.781	-5.028	40.92		6.789	-4.903	40.92	
**3**	-2.468	-0.8092	61.38		-3.198	-2.205	61.38		-3.143	-2.442	61.38	
**4**	2.359	1.1190	81.84		2.671	2.65	81.84		2.498	2.943	81.84	
**5**	-0.9147	-0.1092	102.3		-0.8259	-1.501	102.3		-1.5162	-1.783	102.3	
**6**	-	-	-		-0.3421	1.052	122.76		-0.7691	1.219	122.76	
**7**	-	-	-		0.3757	-0.6214	143.22		0.8433	-0.6394	143.22	
**8**	-	-	-		-0.1669	0.1656	163.68		-0.5873	0.0259	163.68	
**9**	-	-	-		-	-	-		0.3547	0.2456	184.14	
**10**	-	-	-		-	-	-		-0.1265	-0.2224	204.6	
**11**	-	-	-		-	-	-		-0.0154	0.126	225.06	
**12**	-	-	-		-	-	-		0.045	-0.0449	245.52	

* Initial condition
