# Supplementary material for: Resonant model—A new paradigm for modeling an action potential of biological cells
Source: PLoS One. 2019 May 22;14(5):e0216999. doi: 10.1371/journal.pone.0216999 (PMC6530846; doi:10.1371/journal.pone.0216999)
Supplement: S2 Table — (PDF) [file pone.0216999.s006.pdf]

S2 Table. Resonant model (12 subsystems) coefficient values for generating human SAN AP

Subsystem	Integ1 IC*	Integ IC	freq	a_0
**1**	-15.95	-16.900	0.0076	-44.11
**2**	6.86	15.660	0.0152	
**3**	0.7413	-9.6610	0.0227	
**4**	-3.46	3.1850	0.0303	
**5**	1.764	1.5540	0.0379	
**6**	0.7057	-2.0930	0.0455	
**7**	-1.774	0.7472	0.0531	
**8**	1.26	0.7696	0.0606	
**9**	-0.04826	-0.7542	0.0682	
**10**	-0.3961	0.3028	0.0758	
**11**	0.2962	0.2936	0.0834	
**12**	0.07552	-0.2870	0.0910	

***Initial condition**
